# High-resolution methylome analysis uncovers stress-responsive genomic hotspots and drought-sensitive transposable element superfamilies in the clonal Lombardy poplar

**DOI:** 10.1093/jxb/erae262

**Published:** 2024-06-05

**Authors:** Cristian Peña-Ponton, Barbara Diez-Rodriguez, Paloma Perez-Bello, Claude Becker, Lauren M McIntyre, Wim H van der Putten, Emanuele De Paoli, Katrin Heer, Lars Opgenoorth, Koen J F Verhoeven

**Affiliations:** Department of Terrestrial Ecology, Netherlands Institute of Ecology (NIOO-KNAW), Droevendaalsesteeg 10, 6708 PB Wageningen, The Netherlands; Laboratory of Molecular Biology, Wageningen University & Research, 6708 PB Wageningen, The Netherlands; Department of Biology, Philipps-University Marburg, Karl-von-Frisch Strasse 8, D-35043 Marburg, Germany; Eva Mayr-Stihl professorship of Forest Genetics, Albert-Ludwigs-Universität Freiburg, Bertoldstraße 17, 79098 Freiburg i. Br., Germany; Natural Resources and Climate Area, CARTIF Technology Centre, 47151 Boecillo, Valladolid, Spain; IGA Technology Services Srl. Via Jacopo Linussio 51, 33100 Udine UD, Italy; Gregor Mendel Institute of Molecular Plant Biology, Austrian Academy of Sciences, Vienna BioCenter (VBC), 1030 Vienna, Austria; LMU Biocenter, Faculty of Biology, Ludwig-Maximilians-University Munich, 82152 Martinsried, Germany; Department of Molecular Genetics and Microbiology, University of Florida, Gainesville, FL 32611, USA; Department of Terrestrial Ecology, Netherlands Institute of Ecology (NIOO-KNAW), Droevendaalsesteeg 10, 6708 PB Wageningen, The Netherlands; Department of Nematology, Wageningen University & Research, Wageningen 6700 ES, The Netherlands; Department of Agri-Food, Environmental and Animal Sciences, University of Udine, via delle Scienze 206, 33100 Udine, Italy; Department of Biology, Philipps-University Marburg, Karl-von-Frisch Strasse 8, D-35043 Marburg, Germany; Eva Mayr-Stihl professorship of Forest Genetics, Albert-Ludwigs-Universität Freiburg, Bertoldstraße 17, 79098 Freiburg i. Br., Germany; Department of Biology, Philipps-University Marburg, Karl-von-Frisch Strasse 8, D-35043 Marburg, Germany; Biodiversity and Conservation Biology, Swiss Federal Research Institute WSL, Zürcherstrasse 111, CH-8903 Birmensdorf, Switzerland; Department of Terrestrial Ecology, Netherlands Institute of Ecology (NIOO-KNAW), Droevendaalsesteeg 10, 6708 PB Wageningen, The Netherlands; University of Birmingham, UK

**Keywords:** Abiotic stress, biotic stress, differentially methylated region, drought, Lombardy poplar, *Melampsora larici-populina*, *Populus nigra*, short interspersed nuclear element (SINE), whole genome bisulfite sequencing (WGBS)

## Abstract

DNA methylation is environment-sensitive and can mediate stress responses. In trees, changes in the environment might cumulatively shape the methylome landscape over time. However, because high-resolution methylome studies usually focus on single environmental cues, the stress-specificity and long-term stability of methylation responses remain unclear. Here, we studied the methylome plasticity of a *Populus nigra* cv. ‘Italica’ clone widely distributed across Europe. Adult trees from different geographic locations were clonally propagated in a common garden experiment and exposed to cold, heat, drought, herbivory, rust infection, and salicylic acid treatments. Whole-genome bisulfite sequencing revealed stress-induced and naturally occurring DNA methylation variants. In CG/CHG contexts, the same genomic regions were often affected by multiple stresses, suggesting a generic methylome response. Moreover, these variants showed striking overlap with naturally occurring methylation variants between trees from different locations. Drought treatment triggered CHH hypermethylation of transposable elements, affecting entire superfamilies near drought-responsive genes. Thus, we revealed genomic hotspots of methylation change that are not stress-specific and that contribute to natural DNA methylation variation, and identified stress-specific hypermethylation of entire transposon superfamilies with possible functional consequences. Our results underscore the importance of studying multiple stressors in a single experiment for recognizing general versus stress-specific methylome responses.

## Teaser

In poplar, different abiotic and biotic stresses induce methylation changes in common labile genomic regions, while drought induces global CHH hypermethylation, especially over specific transposon superfamilies located close to drought-responsive genes

## Introduction

Plants are constantly challenged by abiotic and biotic stresses that affect their survival, growth, and fitness. Environmental stresses vary in their scale of influence, from temperature variations that may affect the whole plant to pathogens and herbivores that attack plants more locally. Such environmental stimuli can initiate systemic responses that travel to unaffected parts of the plant and trigger acclimation and defense mechanisms (Fichman and Mittler, 2020). At the molecular level, multilayer downstream stress responses are activated, such as complex gene networks, RNA and protein regulation, and epigenetic regulatory mechanisms (Liu and He, 2020). Epigenetic mechanisms are thought to play a crucial role in translating perceived changes in environments to modifications in gene expression and can thus mediate phenotypic plasticity in response to environmental variation.

In plant genomes, DNA methylation is one of the most studied epigenetic modifications and generally refers to the methylation of a cytosine at the fifth carbon position (5mC). Methylation occurs in three sequence contexts: the symmetric CpG and CHG, and the asymmetric CHH context (where H is A, T, or C) (Zhang *et al.*, 2006), which can be inferred at single-base resolution through whole genome bisulfite sequencing (WGBS). DNA methylation plays key roles in transcriptional regulation activity, genome stability (transposon silencing) and genomic imprinting during cell differentiation (Zhang *et al.*, 2018). Moreover, it has been shown that DNA methylation is responsive to environmental factors, and contributes to stress memory (Neves *et al.*, 2017; do Amaral *et al.*, 2020; Liu and He, 2020). Once methylation is established, it can be mitotically heritable depending on the cytosine sequence context (Law and Jacobsen, 2010). Similar to genetic mutations, stable epigenetic variations, or so-called epimutations, can lead to phenotypic variation (Schmitz *et al.*, 2013). The potential for methylation-mediated phenotypic effects is thought to be of particular relevance for long-lived and clonal species to cope with environmental variation (Jueterbock *et al.*, 2020; Miryeganeh *et al.*, 2022).

While the environment-responsiveness of DNA methylation is well established (Liu and He, 2020), its role in mediating environmental plasticity is less well understood, and important questions remain about the nature and functionality of environment-induced DNA methylation (Secco *et al.*, 2015; Bewick *et al.*, 2019; Seymour and Gaut, 2020; Hämälä *et al.*, 2022). For instance, one basic question is whether different environments trigger specific DNA methylation responses, or if most responses reflect a general response. At the functional level, specificity is important for mediating stress-specific stress responses, but general components of stress responses are common (Song *et al.*, 2014; Suzuki *et al.*, 2014; Choudhury *et al.*, 2017; Jia *et al.*, 2017; Gupta *et al.*, 2018; Balfagón *et al.*, 2019; Zandalinas *et al.*, 2020; López Sánchez *et al.*, 2021). Recognizing stress-specific from general responses can help in understanding functionality of the response. However, to date most studies of DNA methylation responses to environments in plants focus on single stresses (but see López *et al.*, 2022), making it difficult to unravel specific from general responses.

*Populus* species have become a model system to study molecular mechanisms in response to environmental cues due to their rapid growth, easy propagation, and available genomic resources (Tuskan *et al.*, 2006; Jansson and Douglas, 2007). Poplars are riparian species that are among the woody plants most sensitive to water stress (Rood *et al.*, 2003; Larchevêque *et al.*, 2011), which has prompted research on molecular mechanisms of drought tolerance (Jia *et al.*, 2016; Viger *et al.*, 2016; Yıldırım and Kaya, 2017), including DNA methylation (Lafon-Placette *et al.*, 2018; Sow *et al.*, 2021). Evidence exists for DNA methylation responses of poplar also to other abiotic and biotic stresses (Song *et al.*, 2016; Zhang *et al.*, 2018; Ashapkin *et al.*, 2020; Xiao *et al.*, 2021; Hannan Parker *et al.*, 2022), and plant hormones (Xuan *et al.*, 2020). However, because of the use of low-resolution DNA methylation screening methods and because experiments typically focus on single-stress treatments, insight in environmental DNA methylation plasticity in this model system remains fragmented. Evaluating and comparing methylation patterns in poplar induced by both abiotic and biotic factors, along with their long-term stability, will increase our understanding of generic versus stress-specific methylation responses, and might provide new clues to how long-lived species acclimate to simultaneous stresses in nature.

Here we use a common garden experimental approach to characterize the DNA methylation response of a clonal cultivar of poplar when exposed to a range of different experimental treatments that mimic ecologically relevant biotic and abiotic stresses. We took advantage of a long-term natural experiment where a single clonal lineage of Lombardy poplar (*Populus nigra* cv. ‘Italica’) had been planted in different European locations, so that we capture the methylation variation that has been built up historically between genetically homogeneous trees during their lifetime of growth in different environments (Díez-Rodríguez *et al.*, 2022, Preprint). We clonally propagated replicate individuals of eight trees from different locations, and in a controlled greenhouse experiment, we exposed the propagated trees to different experimental environments (control, cold, heat, drought, rust infection, caterpillar herbivory and salicylic acid signaling). We addressed the following research questions: (i) to what extent are DNA methylation responses stress-specific? (ii) how do methylome responses to stress compare with methylation variation observed between trees? and (iii) what are the specific features of common and stress-specific methylation responses and how do these relate to potential functionalities?

## Materials and methods

**Fig. 1.**
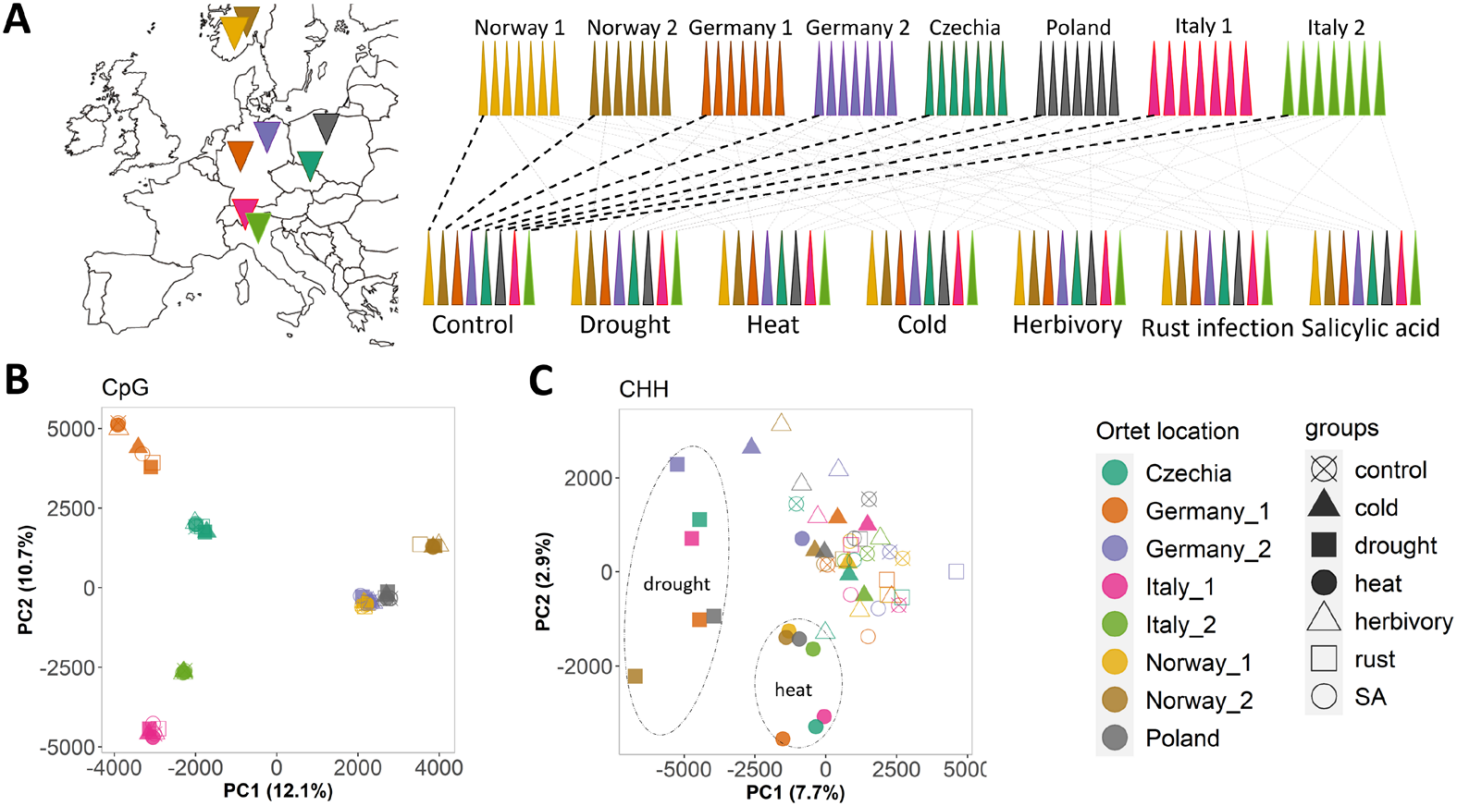
Analysis of genome-wide methylation patterns from stress-treated Lombardy poplar ramets. (A) Sampling locations of the clonally propagated ortets and their ramet representation in experimental groups. (B, C) Unsupervised principal component analysis of CpG and CHH methylation. Ramets are colored according to their ortet identity. Different shapes represent distinct experimental groups. Drought and heat clusters are highlighted with dashed ovals. SA, salicylic acid.

We used plant material and genomic resources developed under the scope of the EpiDiverse Innovative Training Network (https://epidiverse.eu/). Collection of the poplar samples from different countries is described in Díez-Rodríguez *et al.* (2022, Preprint), and a reference genome of the *Populus nigra* cv. ‘Italica’ genotype under study was generated in the EpiDiverse project (www.ebi.ac.uk/ena/browser/view/GCA_950102115). We leveraged this infrastructure in a common garden experiment that exposed replicate ramets from each of eight ortets from different geographic locations to each of six different stress conditions plus control group (Fig. 1A).

### Plant material

Between February and April 2018, hardwood cuttings (approximately 30 cm length) from adult *Populus nigra* cv. ‘Italica’ clones (ortets hereafter) were collected across Europe (Díez-Rodríguez *et al.*, 2022, Preprint) and were initially stored at 4 °C for 2 weeks and then planted in 4-liter pots with a 3:1 sand:peat mixture (v/v) and placed on a flood table for rooting. All ortets used in this study were confirmed to belong to the same clonal lineage through Allegro genotyping (Díez-Rodríguez *et al.*, 2022, Preprint). Low levels of somatic mutation variation exist between clone members [on average <100 single nucleotide polymorphisms (SNPs) between two samples among a panel of >8000 informative SNP positions screened; Díez-Rodríguez *et al.*, 2022, Preprint]. Such genetic variation, albeit very low, can in principle be responsible for some DNA methylation differences between clone members (Becker *et al.*, 2011; Dubin *et al.*, 2015). To be able to account for location-specific methylation profiles from the source environment (Raj *et al.*, 2011; Schönberger *et al.*, 2016; Heer *et al.*, 2018), we included ramets derived from eight different ortets (Supplementary Table S1) in each of the seven stress treatments (cold, heat, drought, rust infection, caterpillar herbivory, and salicylic acid signaling, compared with a control group) leading to a total of 56 similar-height ramets (Fig. 1A; Supplementary Fig. S1). By having each ortet contribute one replicate plant to each of the stress treatments, possible methylation differences between ortets are not confounded with experimental treatment effects, resulting in a clean test of treatment effects on DNA methylation across a potentially epigenetically diverse set of ortets. The ramets were transferred to 7 liter pots with a 1:1 sand:peat mixture and maintained with regular watering under controlled conditions for a total of 12 weeks until the start of the experiment. All treatments were implemented simultaneously during a period of 25 d, which included two 10 day stress events and one 5 day stress-free period in between (Supplementary Fig. S1). The experiment was laid out in a Latin square (Supplementary Fig. S2). Growth conditions were: 22/18 °C (±2 °C) at day/night, 60% relative humidity (±5%), and 16/8 h light/dark. Two weeks prior to the start of the experiment, 3 g of slow-release fertilizer, Osmocote Exact Mini (16 + 8+11 + 2MgO+TE), was added to each pot. Here, we provide an essential overview of the experimental methods; more detailed information can be found in Peña-Ponton *et al*. (2023; https://zenodo.org/record/8428770).

#### Control group

During the stress experiment, control plants were maintained in a greenhouse under the conditions above. The soil volumetric water content (VWC) was maintained on average at 20.2% (±3.24 SD) by daily watering to pot capacity. VWC was monitored daily using the WET Sensor kit (Delta-T Devices). Mean VWC was calculated for all plants with two measurements per pot (Supplementary Fig. S3A).

#### Biotic stresses

Rust infection consisted in spray-inoculation of uredospores of the poplar leaf rust fungus (*Melampsora larici-populina* Kleb.). Herbivory treatment involved the use of gypsy moth caterpillars (*Lymantria dispar* L.). Salicylic acid (SA) treatment consisted in spray application of 1 mM SA (Sigma-Aldrich). Rust spores and caterpillars were obtained from Dr Sybille Unsicker (Christian-Albrechts-University of Kiel, formerly at Max Planck Institute for Chemical Ecology, Jena, Germany).

#### Abiotic stresses

Drought stress was attained by withholding watering and maintaining VWC at 8%. Plants that received heat treatment were grown at high temperatures of 30–38/28 °C (day/night), while cold-treated plants were grown at 4/4 °C (day/night). VWC for all treatments (except drought) was maintained close to control conditions (Supplementary Fig. S3A). Plants were moved to climate chambers for heat and cold treatments (Supplementary Fig. S3B). During the stress-free period, plants were moved to the same greenhouse table as the control group and placed using the same Latin square layout (Supplementary Fig. S2).

#### Harvesting

Many of the studies on the effect of different abiotic and biotic stresses in poplar have reported DNA methylation changes in young mature leaves (Raj *et al.*, 2011; Liang *et al.*, 2014; Schönberger *et al.*, 2016; Xuan *et al.*, 2020). Therefore, in order to conduct a reasonable comparison among the diverse treatments used in this study, on experimental day 26, we sampled fully expanded young leaves that developed during the stress experiment. Twelve circular punches (Ø 8 mm; ~100 mg fresh weight in total) were cut out from the eighth mature leaf (counting from the apex of the main branch, leaf plastochron index: 10) of each plant. Mid-ribs were avoided, and leaf punches were immediately frozen in liquid nitrogen and stored at −80 °C. We aimed to compare systemic-related methylation responses as we sampled leaves that were not directly exposed to any stress, with the exception of cold and heat. All sampled leaves showed no signs of damage/infection at the time of sampling. Ten days after the end of the experiment, all ramets were coppiced. Stems and leaves were dried at 70 °C for 7 d, then dry weight biomass was determined. All plants were maintained with minimal watering from the end of the experiment until coppicing to avoid further growth.

### DNA extraction and whole genome bisulfite sequencing

Per sample, frozen leaf tissue was ground and homogenized using a TissueLyser II (Qiagen), then genomic DNA was isolated using the SDS procedure of the NucleoSpin Plant II DNA isolation kit (Macherey-Nagel, Dueren, Germany). Preparation of DNA libraries for bisulfite sequencing was performed as described in Nunn *et al.* (2022). All sequencing was performed by Novogene on an Illumina HiSeq X Ten sequencing system. Libraries were sequenced with 2 × 150 bp paired-end reads at 30× coverage.

### Processing of bisulfite-treated reads and methylation calling

Sequenced reads were processed using the EpiDiverse Toolkit (WGBS pipeline v1.0, https://github.com/EpiDiverse/wgbs) (Nunn *et al.*, 2021). Briefly, low-quality read-ends were trimmed (minimum base quality: 20), sequencing adapters were removed (minimum overlap: 3 bp), and very short reads (<36 bp) were discarded. The remaining high-quality reads were aligned to the *Populus nigra* var. ‘Italica’ *de novo* reference genome (www.ebi.ac.uk/ena/browser/view/GCA_950102115) using erne-bs5 (http://erne.sourceforge.net) allowing for 600 bp maximum insert size, 0.05 mismatches, and unique mapping. All cytosines with low sequencing coverage (≤5 reads) were removed from consideration. Per-cytosine methylation metrics were calculated using MethylDackel (https://github.com/dpryan79/MethylDackel). The percentage methylation per-cytosine was recorded:


$$\begin{array}{l} \frac{Methylatedcytosinereadcount}{Methylatedcytosinereadcount+Unmethylatedcytosinereadcount}\times 100 \end{array}$$

For each sample, three bedGraph files corresponding to each sequence context (CpG, CHG, CHH) were obtained. Then, we produced three bedGraph files, one for each sequence context, containing information from all samples.

### Methylation analysis

The analysis of methylation was separated into (i) genome-wide methylation analysis to detect global methylation patterns, and (ii) differential methylation analysis to identify differentially methylated regions (DMRs). The three cytosine sequence contexts were analysed separately. Data filtering and resolution was slightly different for each analysis (Supplementary Table S2).

#### Average global methylation

For the genome-wide methylation analysis, we excluded samples in which the retained cytosines accounted for less than 50% of the original data. These low-coverage samples were excluded from genome-wide analyses unless otherwise stated (4 out of 56 samples). Cytosines with sufficient coverage across all 52 samples that remained after filtering were analysed (CpG: 1 802 288 positions, CHG: 3 256 938 positions, CHH: 12 058 984 positions). For each sample, average global methylation (%) was calculated for each context. The effect of the stress treatments on average global methylation was evaluated for each context using a linear mixed model with treatment as fixed factor and parental tree (ortet) as random factor. The effect of the ortet was evaluated in the same manner. Statistical analyses were calculated in R (version 4.0.3); the *lmer* function of the *lmerTest* package (Kuznetsova *et al*., 2017) was used to fit the model; and multiple comparisons (Tukey’s post-hoc test) were calculated with the *glht* function of the *multcomp* package (Hothorn *et al.*, 2008).

#### Multivariate analyses of samples

Principal component and hierarchical clustering of samples was performed based on percentage methylation for individual cytosines in the CpG and CHG context. For CHH there were a number of invariant cytosines (more than 95% or less than 5% methylation in all samples) that were removed. Principal components (PCs) were calculated in R using the *prcomp* function of the *stats* package (R Core Team, 2022). Hierarchical clustering (HC) (Ward’s method) was computed by first calculating the corresponding distance matrix (Manhattan method) using the *dist* and *hclust* functions from the *stats* R package. Intraclass correlation analysis was performed using genomic regions instead of single positions. The poplar genome was compartmentalized in 100 bp non-overlapping bins. Average methylation per bin was calculated, and only bins with informative methylation information across all samples were retained. We calculated the intraclass correlation coefficient (ICC) for all pairwise comparisons between samples, as this is a measure of agreement between samples and accounts for within sample as well as between samples variation (Fleiss and Cohen, 1973; Koo and Li, 2016). Coefficients were calculated in R using the *icc* function of the *irr* package (Gamer *et al.*, 2019) with the ICC form: two-way random effects, absolute agreement, single measurement, according to (McGraw and Wong, 1996) convention.

#### Methylation profiles

All (56) samples were included in the analysis. For gene regions, only protein-coding genes with known 5ʹ untranslated region (UTR) and 3ʹUTR coordinates were considered. For transposable elements (TEs), only those longer than 200 bp were analysed. Methylation profiles over the largest poplar scaffold (scaffold 1=33 746 648 bases) were used as a proxy for chromosome-wise methylation variation comparison. The scaffold was compartmentalized in 50 kb bins, then for each sample, per-bin average methylation was calculated. Per-bin methylation for each sample was calculated as the average value weighted by the number of cytosines in the bin (Cochran, 1977). For each cytosine context, the per-bin methylation difference between each treatment and the control group was calculated and used to plot heatmaps and simple moving averages. Simple moving averages were calculated using the R function *geom_ma* of the *tidyquant* package (Dancho, 2022).

#### Differentially methylated region calling

DMRs induced by each stress treatment were identified by testing local methylation differences between each treatment and control group. All replicates per treatment were included in the tests and each cytosine sequence context was analysed separately using the EpiDiverse/DMR pipeline v0.9.1 (https://github.com/EpiDiverse/dmr) (Nunn *et al.*, 2021). Briefly, DMRs were identified by metilene (https://www.bioinf.uni-leipzig.de/Software/metilene), with parameters as follows. Minimum read depth per position: 6; minimum cytosine number per DMR: 10; minimum distance between two different DMRs: 146 bp; per-group minimal non-missing data for estimating missing values: 0.8; adjusted *P*-value (Benjamini and Hochberg, 1995) to detect significant DMRs: 0.05. Only significant DMRs with minimum methylation difference of 10 percentage points between groups were used for downstream analyses.

Since the genome-wide methylation analyses showed strong CpG and CHG methylation patterns associated with ortets irrespective of stress treatment, stress-DMRs were identified using a jack-knife approach (leave-one-out) to reduce the influence of individual ortets (Supplementary Fig. S4). Genomic coordinates of DMRs appearing in two or more DMR callings were merged using *bedtools merge* (minimum overlap of 1 bp). Control-treatment methylation difference, length, and cytosine number for each merged DMR were calculated by averaging the corresponding values of the merged DMRs (summary statistics are shown in Supplementary Table S3). Additionally, to check if our jack-knife approach for DMR calling produced results consistent with other approaches, we called stress-DMRs using Methylkit (Akalin *et al.*, 2012). There was a large overlap between both methods with our approach being generally more conservative (Supplementary Table S4). DMRs that were identified in more than one treatment were classified as multi-stress DMRs. Genomic regions where a DMR was identified in more than one sequence context were labelled as multi-context DMRs.

We also called DMRs among ortets, considering ramets derived from the same ortet as replicates irrespective of their exposure to different treatments. Briefly, DMRs were called for all pairwise comparisons among the eight ortets (total: 28 DMR-sets per context; summary statistics are shown in Supplementary Table S5). Next, for each context, all DMRs were classified according to the number of pairwise comparisons in which each DMR appeared in: DMRs found in a *unique comparison* or DMRs *shared by two or more comparisons*. Intersections between stress-DMRs and ortet-DMRs were performed using bedtools *intersect*.

#### DMR annotation

Statistically significant DMRs were annotated using the *Populus nigra* cv. ‘Italica’ protein-coding gene model annotation (Perez-Bello Gil and De Paoli, 2023). Only the longest transcript per gene was used for this analysis. DMRs were also associated with TEs based on a TE prediction for this cultivar (https://zenodo.org/record/8428770). Gene models and TE predictions used in this study were generated as part of the ongoing *P. nigra* cv. ‘Italica’ reference genome project (www.ebi.ac.uk/ena/browser/view/GCA_950102115). Short descriptions of these annotation files can be found along with their deposited versions. Short interspersed nuclear elements (SINEs) were manually added to the predicted TEs based on BLASTN results (70% similarity, 90% coverage) using the consensus sequences of *Salicaceae* SINE families (Kögler *et al.*, 2020).

#### DMR enrichment on genomic features

For each context, all DMRs, irrespective of treatment, were tested for enrichment in gene bodies, exons, introns, gene flanking regions and TEs (*Z*-test for proportions). As DMRs were enriched in TEs, we also tested whether the occurrence of DMRs in gene bodies, exons, introns, and gene flanking regions was conditional on the presence of TEs (chi-square tests for independence, McNemar’s test; McNemar, 1947). Additionally, as drought showed the largest response associated to TEs, we calculated the relative fold enrichment for drought CHH-DMRs on each TE superfamily. *P*-values were obtained from the hypergeometric test (Falcon and Gentleman, 2008) and then adjusted (Bonferroni) according to the number of TE superfamilies tested. Drought-DMR-enriched TE superfamilies were referred to as drought-responsive TEs (DR-TEs), which included all SINE and miniature inverted-repeat transposable elements (MITE), especially MITE/DTH (Harbinger-related) elements.

#### Generic (multi-stress) versus stress-specific methylation responses

Since stress-DMRs were identified between each treatment versus the control group, we were interested in evaluating the methylation response of such regions induced by all the other treatments. In addition to identifying DMRs that were statistically significant in more than one stress treatment (multi-stress DMRs, see above), we calculated average methylation levels of the corresponding regions on each sample using the genomic coordinates of each identified DMR. Then, we calculated the average methylation level across treatment replicates. Finally, for each region, we computed the methylation difference between each treatment and control group. We only analysed regions with enough methylation information (≥8 cytosines) and replication (≥6 replicates).

#### Drought-specific transposable element analysis

As we detected TE superfamilies enriched with drought-DMRs, we analysed the drought-induced methylation response of all poplar TE superfamilies to check for generalized responses of entire TE superfamilies. For each sequence context, average methylation levels of each individual TE were calculated for drought and control samples. We only analysed TEs with enough methylation information (≥20 cytosines for CHH, ≥10 cytosines for CpG and CHG) and replication (≥6 replicates). For each TE element, we computed the methylation difference between drought and control group. Then, to summarize and compare results among TE superfamilies, we grouped TE elements in boxplots according to each superfamily.

#### Gene ontology enrichment analysis

Functional enrichment analysis had to be carefully interpreted as gene expression data were not collected in this experiment and most stresses produced very few DMRs. Therefore, our analysis was mainly focused on medium-to-large gene sets associated with drought-induced methylation responses.

Genes associated with drought CHH-DMRs were subjected to gene ontology (GO) enrichment analysis. The gene background was built with the closest Arabidopsis homologue of each *P. nigra* cv. Italica gene, which was determined using BLAST reciprocal best hits (RBH) of the protein sequences (R package *orthologr*; Drost *et al.*, 2015). Best hits were filtered by keeping alignments covering at least 60% of both Arabidopsis and *P. nigra* proteins, and minimum 60% similarity. Arabidopsis protein sequences were extracted from phytozome V13, and functional annotations were retrieved from the PLAZA 5.0 dicots database (https://bioinformatics.psb.ugent.be/plaza/). GO enrichments were performed using clusterProfiler v4 (Wu *et al.*, 2021), a tool designed to perform over-representation analysis (Boyle *et al.*, 2004). *P*-values were adjusted for multiple testing controlling the positive false discovery rate (*q*-value) (Storey, 2002).

Enrichments for genes associated with all SINE and MITE/DTH elements were performed in the same manner. Gene sets for functional enrichment included genes associated with either all SINEs, or all MITE/DTHs, or both (DR-TEs). Additional enrichments were performed for a subset of potential highly drought-responsive TEs (HDR-TEs), i.e. SINEs and MITE/DTHs that displayed at least 5% hypermethylation compared with the control group. Finally, for comparison, all enrichments were analysed in the context of the drought CHH-DMR gene set enrichment.

## Results

### Drought induces a large and distinctive genome-wide CHH hypermethylation response in the Lombardy poplar

Genome-wide methylation analyses were performed to evaluate methylation patterns among ortets and treatments. Linear mixed models revealed significant treatment effects on average global DNA methylation (CpG: *P*=0.048; CHG: *P*=0.043; CHH: *P*<0.01). However, only drought treatment induced a significant global increase of CHH methylation compared with control group (Tukey’s test, *P*<0.01). In addition, cold treatment induced significantly higher CpG and CHG methylation levels compared with salicylic acid treatment (*P*=0.0241 and 0.0412, respectively) (Supplementary Fig. 2A). Both drought and cold treatments resulted in reduced growth after the treatment (Supplementary Fig. S5).

**Fig. 2.**
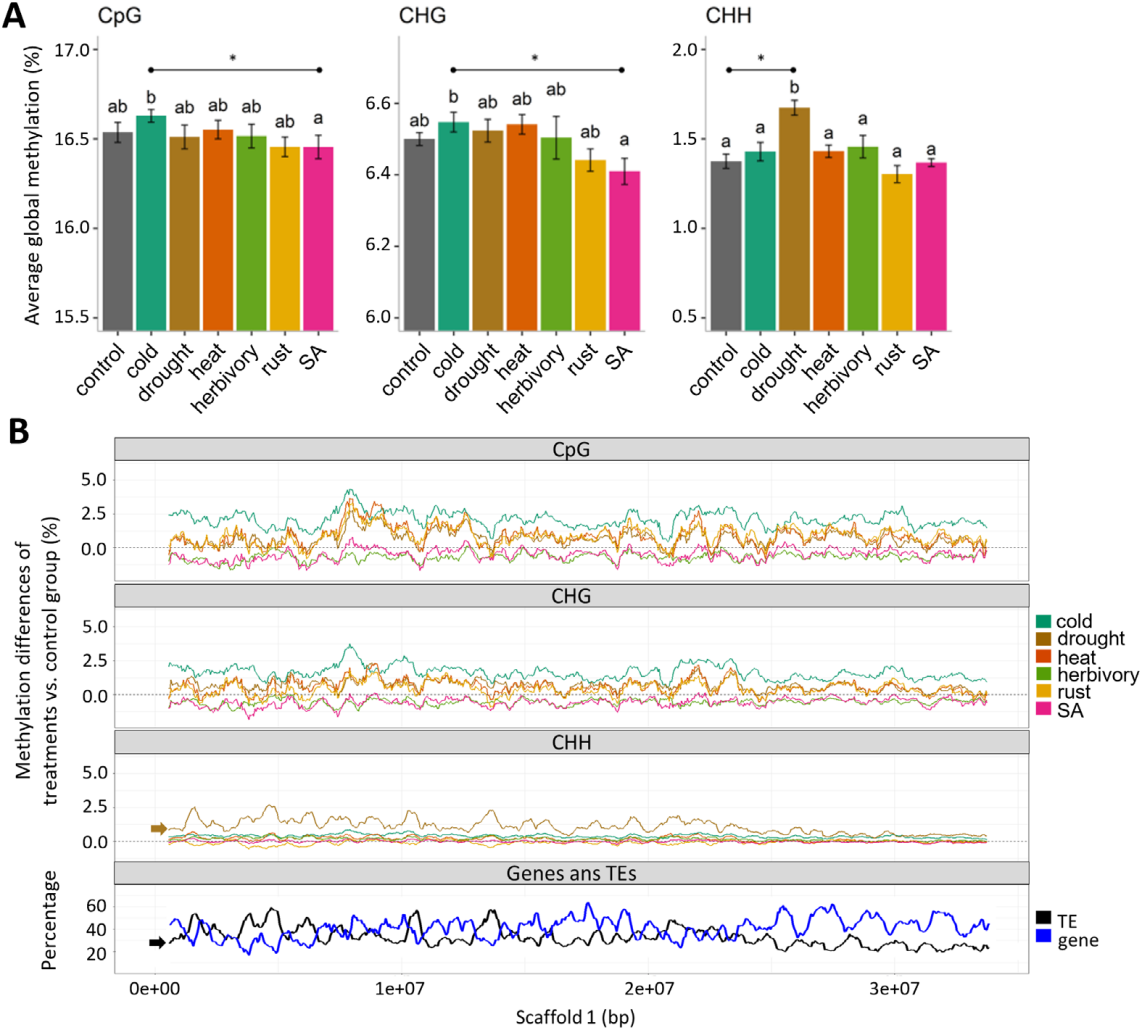
Stress-induced genome-wide DNA methylation variation in the Lombardy poplar. Metaplots of methylation level differences (treatments versus control group) and gene/transposable element (TE) content over the scaffold 1 of *Populus nigra* var. Italica. (A) Bar plots of average global methylation (%) for each ortet. Sequence contexts were analysed separately. Horizontal bars and letters indicate relevant significant pairwise differences after Tukey post-hoc comparisons. The values are the means ±SE; *n*=8. (B) Metaplots of methylation level differences (treatments versus control group) and gene/TE content over the scaffold 1 of *Populus nigra* var. Italica. Top panels: scaffold 1 was divided into 50 kb bins, per-bin methylation differences for each treatment and context (CpG, CHG, CHH) versus control group were calculated, then simple moving averages (SMA) over a period of ten 50 kb bins was plotted. Bottom panel: profiles for TE and gene content for 50 kb bins (SMAs per 10 bins). Arrows highlight the relationship between methylation variation and gene/TE content, especially obvious for drought-induced CHH methylation variation (brown arrow) and TE content profile (black arrow). SA, salicylic acid.

Principal component analysis (PCA) and HC analyses of CHH methylation highlighted noticeable clusters for drought and heat treatments (Fig. 1C; Supplementary Figs S6C, S7C). High correlations were observed among drought-treated samples, while the lowest correlation coefficients were found when comparing drought samples with any other sample (Supplementary Fig. S8C). Methylation profiles over the largest poplar scaffold confirmed the genome-wide drought-induced CHH hypermethylation and underlined a close relationship with TE content as both profiles showed peak similarities (Fig. 2B). Over TE regions, profiles corroborated the drought-induced CHH hypermethylation (Supplementary Fig. S9), and highlighted CHH hypomethylation induced by rust infection (Supplementary Fig. S10). Profiles over genic regions revealed that drought-induced CHH hypermethylation mainly targeted gene-flanking regions rather than gene bodies (Supplementary Figs S10, S11).

The effect of other treatments was also observed in the methylation profiles. Profiles of CpG/CHG methylation along the scaffold 1 confirmed the genome-wide cold-induced hypermethylation that was already detected in the global methylation analysis. In addition, several treatments showed overlapping profiles of hypermethylation (drought, heat, and rust), and hypomethylation (SA and herbivory). Visual observation of the methylation profiles indicated a positive correlation between CpG and CHG methylation variation (Fig. 2B; Supplementary Fig. S9). Interestingly, over genic regions, the effects of stress treatments were detectable mainly in gene flanking regions while in TE regions, cold and SA induced the largest CpG/CHG methylation responses: hypermethylation and hypomethylation, respectively (Supplementary Fig. S10).

### Genome-wide CpG and CHG methylation largely reflect sample origin

Linear mixed models revealed significant ortet effects on the average global CpG methylation (*P*<0.01) (Supplementary Fig. S12). Detailed insights were observed on PCA, HC, and correlation analysis where distances among ramets derived from the same ortet (within-ortet) were much smaller than those among ramets derived from different ortets (between-ortet), irrespective of the treatment (Fig. 1B; Supplementary Figs S6A, S8A, D). A similar but less pronounced within-ortet clustering was found for CHG methylation, with an additional clustering of drought‐treated ramets, irrespective of the ortet (Supplementary Figs S6–S8).

### Transposable elements are enriched with stress-induced differentially methylated regions

**Fig. 3.**
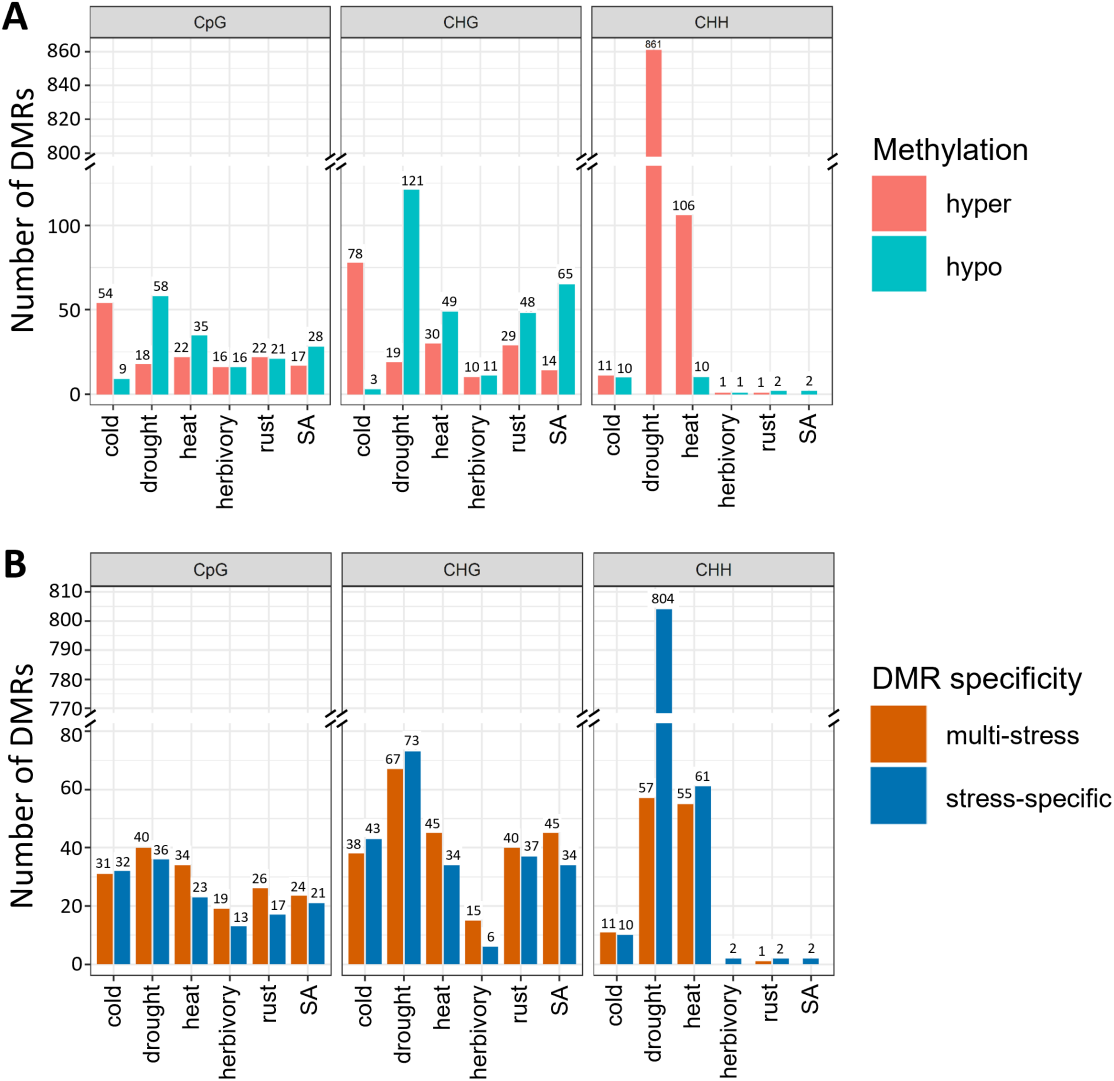
Summary of significant differentially methylated regions (DMRs) induced in the Lombardy poplar by each stress treatment versus the control group in CpG, CHG, and CHH contexts. (A) DMRs classified by methylation direction: hypermethylated (red) and hypomethylated (blue). (B) DMRs classified by specificity: multi-stress (orange) and stress-specific (dark blue). DMR numbers are shown on top of each bar. SA, salicylic acid.

We identified a total of 1798 DMRs across all treatments and sequence contexts (Fig. 3; Supplementary Table S6). Drought induced the largest number of DMRs among all treatments (mostly CHH hypermethylations), while cold induced the largest amount of hypermethylated DMRs in CpG/CHG contexts (Fig. 3A; Supplementary Table S6). In general, similar amounts of multi-stress and stress-specific DMRs were observed in each treatment, except for drought CHH-DMRs (Fig. 3B; Supplementary Table S7). Among the 203 multi-stress DMRs, drought and heat showed the largest intersection with 57 DMRs (Supplementary Fig. S13).

**Fig. 4.**
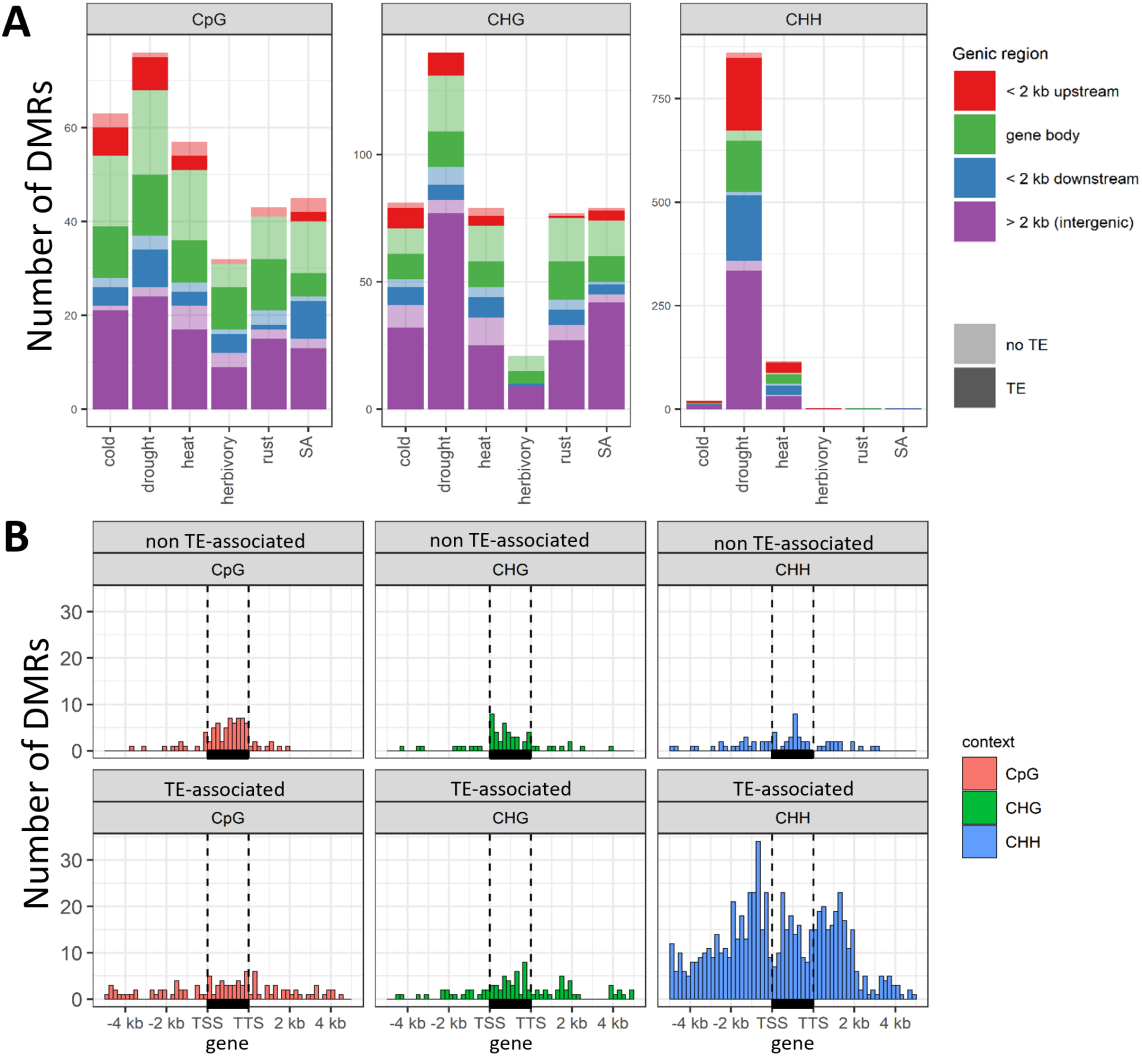
Distribution of stress-induced differentially methylated regions (DMRs) over the Lombardy poplar genome. (A) For each treatment and context, DMR counts (irrespective of treatment) are shown for gene body (including exons, introns and untranslated regions), ±2 kb gene flanking regions, and intergenic regions. Dark/light colors differentiate the number of DMRs associated/not associated with transposable elements (TEs) in the corresponding region. (B) Detailed distribution of all stress-induced DMRs along genic regions, per context and TE association. Vertical dashed lines indicate the gene transcription start site (TSS) and transcription termination site (TTS). Horizontal black boxes represent the gene body. Gene lengths were normalized to 2 kb. SA, salicylic acid.

Enrichment tests showed that all DMRs, irrespective of sequence context, were enriched in TEs (Supplementary Table S8). In addition, CpG-DMRs mainly targeted gene bodies, specifically exons, while CHH-DMRs were enriched over gene flanking regions (Fig. 4B; Supplementary Table S8). CHG-DMRs were enriched in intergenic regions associated with TEs, while TE-associated CHH-DMRs were enriched in gene flanking regions and introns (Supplementary Table S9). Moreover, we observed an increased frequency of DMRs in TE flanking regions, especially within the first 200 bp (Supplementary Fig. S14), revealing TEs as a major source of methylation variation (DMRs) irrespective of treatment (Supplementary Figs S15, S16).

### Drought-induced CHH hypermethylation is stronger in specific transposable element superfamilies

**Fig. 5.**
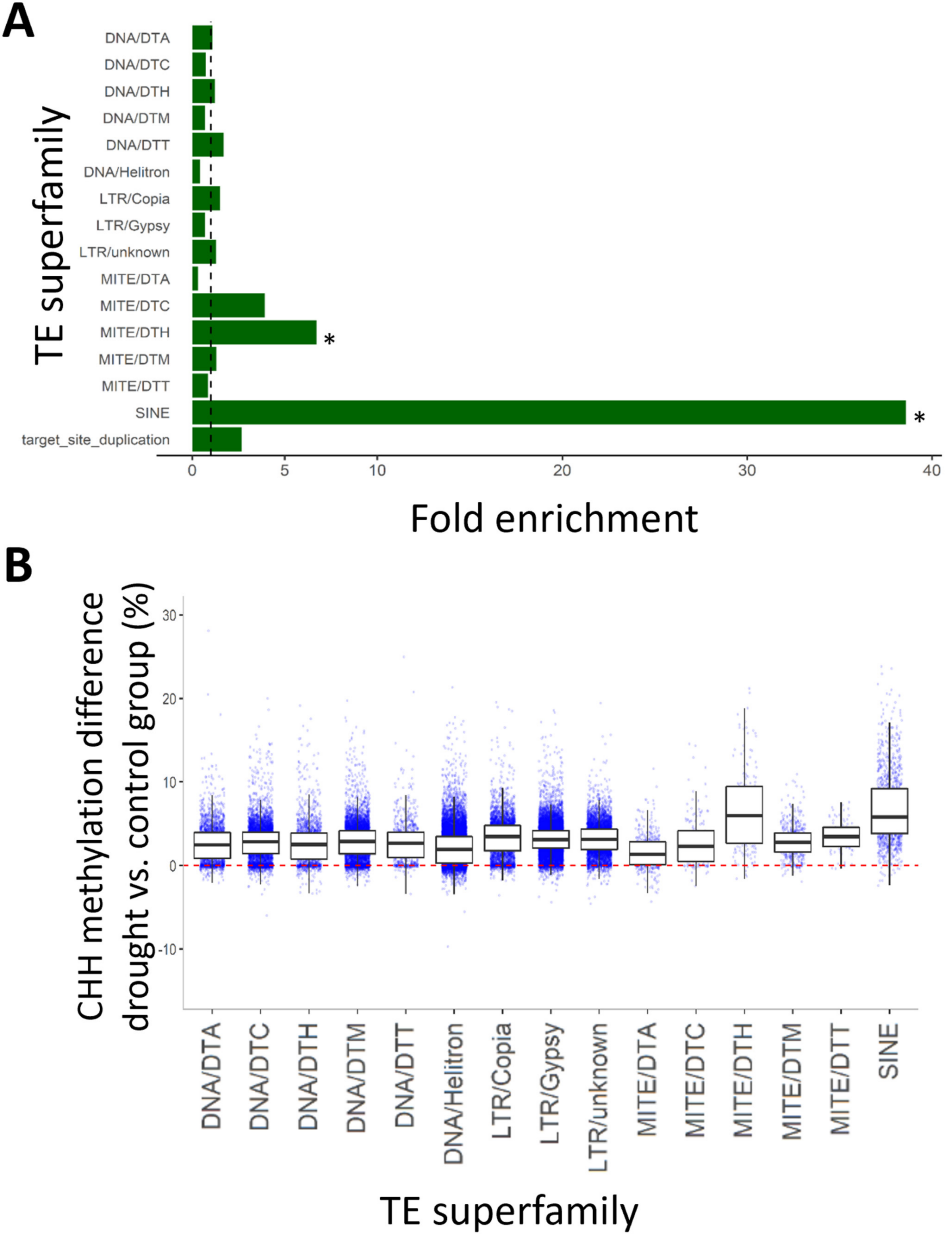
Analysis of drought-induced CHH hypermethylation of the Lombardy poplar transposable element (TE) superfamilies. (A) Fold enrichment analysis of TE superfamilies targeted by drought CHH differentially methylated regions (DMRs). Enrichments were calculated based on the total length of each TE superfamily in the genome. Hypergeometric tests identified significant enrichments (*P*<0.001) for short interspersed nuclear element (SINE) and Harbinger-related miniature inverted-repeat transposable element (MITE/DTH) superfamilies (Supplementary Table S10). (B) Boxplots of drought-induced CHH methylation variation (versus control group) over individual TE elements. Each boxplot summarizes the overall methylation response of a specific TE superfamily (*x*-axis) to drought stress. The horizontal dotted red line depicts the zero drought-control difference. Based on their terminal inverted repeats and tandem site duplications sequences, TEs were assigned to different superfamilies represented by different codes with DTA for *h*AT, DTC for *CACTA*, DTH for *PIF/Harbinger*, DTM for *Mutator*, and DTT for *Tc1/Mariner*.

DMR enrichments over each TE superfamily revealed that SINE and MITE, especially MITE/DTH, showed an exceptionally strong response (Fig. 5; Supplementary Table S10). Interestingly, SINE and MITE/DTH elements also display the highest CHH methylation under control conditions among all TE superfamilies (Supplementary Fig. S17). Methylation analysis over genic regions showed that drought induced CHH hypermethylation of SINE and MITE/DTH elements irrespective of gene proximity (Supplementary Figs S18, S19, respectively).

### CpG/CHG stress differentially methylated regions are also multi-stress differentially methylated regions and ortet differentially methylated regions

**Fig. 6.**
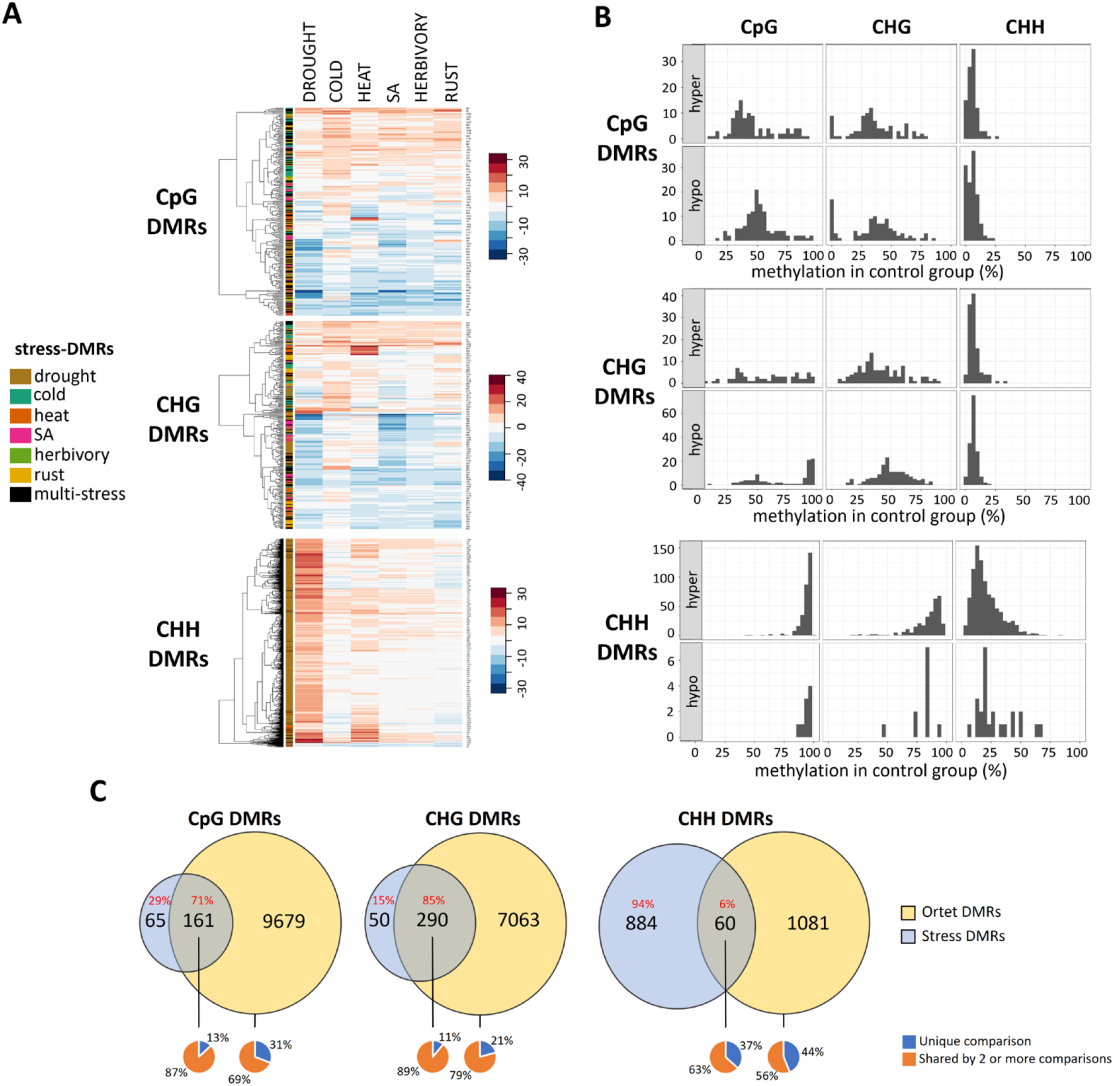
Patterns of differentially methylated regions (DMRs) identified in the Lombardy poplar. (A) Heatmap and hierarchical clustering of the average difference methylation levels (compared with control) of the 1728 identified stress-DMRs. (B) Histograms of CpG, CHG, and CHH methylation level in the control group for all stress-DMRs. Histograms are shown according to DMR features (context and response: hyper/hypo). (C) Venn diagrams of the intersections between ortet-DMRs and stress-DMRs for each sequence context. The uniqueness of ortet-DMRs (and for the intersection) is shown below the Venn diagrams. SA, salicylic acid.

By examining the methylation response of stress-specific DMR regions in all other treatments, we observed that most of the stress-specific CpG/CHG-DMRs also showed a response to other treatments, usually in the same direction (either hyper- or hypo-methylation) (Fig. 6A; Supplementary Fig. S20). Thus, different stresses tended to result in similar methylation responses at these genomic locations, even when statistical significance was only reached in response to some treatments. Moreover, by examining the methylation level of these responsive regions in the control group, we noticed that CpG/CHG-DMRs had intermediate CpG/CHG methylation and low CHH methylation, while CHH-DMRs showed very high methylation in all contexts (Fig. 6B).

The amount of ortet-DMRs was several orders higher than the stress-DMRs, especially in the CpG/CHG context. For each comparison between two ortets, we identified on average 1425 CpG-DMRs, 1621 CHG-DMRs, and 133 CHH-DMRs (Supplementary Fig. S21). We detected a total of 9840 CpG-DMRs, 7353 CHG-DMRs and 1141 CHH-DMRs after accounting for DMRs found in more than one pairwise comparison (Supplementary Table S11). Such ortet-DMRs were considered as a product of natural methylation variation, and when intersected with stress-DMRs, the analysis revealed that most of the stress CpG-DMRs (71%) and CHG-DMRs (85%) were also identified as ortet-DMRs. However, only 6% of stress CHH-DMRs were found in the ortet-DMR set. (Fig. 6C; Supplementary Table S11).

### Functional analysis of genes associated to drought differentially methylated regions and drought-responsive transposable elements

Enrichment analyses revealed few gene ontology (GO) terms that were significant after multiple testing correction (*q*-value). GO terms with uncorrected *P*-values (<0.05) suggested that genes associated with drought CHH-DMRs were enriched in processes related to response to abiotic stimulus (GO:0071214), osmotic stress (GO:0006970), and water deprivation (GO:0009414). Comparisons of functional enrichments of gene sets associated to drought CHH-DMRs and drought-responsive TEs (DR-TEs) highlighted considerable overlaps. Response to abscisic acid (GO:0009737) and protein kinase activity (GO:0004672) were terms that were enriched in almost all gene sets, while cellular response to water deprivation (GO:0042631) and cellular response to water stimulus (GO:0071462) were enriched only in drought-DMR, MITE/DTH, and DR-TE sets. In addition, SINE-associated genes were mainly enriched in terms related to protein phosphorylation while MITE/DTH-associated genes were mostly enriched in terms related to abscisic acid (ABA)/hormone signaling and response to water stimulus. Gene sets associated with HDR-TEs showed similar enrichments to those accounting for DR-TEs (Table 1). Thus, regions and TE superfamilies that showed methylation responses to drought seem to be located close to drought-responsive genes.

**Table 1.** Gene Ontology (GO) enrichment analysis of genes associated with drought CHH-DMRs and drought-responsive TEs (DR-TEs) and highly drought-responsive TEs (HDR-TEs)

GO ID	GO description	Ontology	DR-TEs			HDR-TEs (5% hypermethylation)			Drought CHH-DMRs
			SINEs	MITE/DTH	both	HDR SINEs	HDR MITE/DTH	Both	
GO:0009719	Response to endogenous stimulus	BP		×	×				×
GO:0009725	Response to hormone	BP		×	×				×
GO:0016310	Phosphorylation	BP	×		×	×		×	×
GO:0006468	Protein phosphorylation	BP	×		×	×		×	×
GO:0001101	Response to acid chemical	BP			×				×
GO:0097305	Response to alcohol	BP	×	×	×	×	×	×	×
GO:0009737	Response to abscisic acid	BP		×	×	×	×	×	×
GO:0071229	Cellular response to acid chemical	BP		×	×				×
GO:0042631	Cellular response to water deprivation	BP		×	×				×
GO:0071462	Cellular response to water stimulus	BP		×	×				×
GO:0019853	l-Ascorbic acid biosynthetic process	BP	×		×				×
GO:0009963	Positive regulation of flavonoid biosynthetic process	BP					×		×
GO:0019632	shikimate metabolic process	BP						×	×
GO:0016772	Transferase activity, transferring phosphorus-containing groups	MF	×			×		×	×
GO:0016301	Kinase activity	MF	×			×		×	×
GO:0016773	Phosphotransferase activity, alcohol group as acceptor	MF	×		×	×		×	×
GO:0004672	Protein kinase activity	MF	×		×	×		×	×
GO:0004674	Protein serine/threonine kinase activity	MF	×		×	×		×	×
GO:0106310	Protein serine kinase activity	MF	×		×	×		×	×
GO:0106311	Protein threonine kinase activity	MF	×		×	×		×	×
GO:0050660	Flavin adenine dinucleotide binding	MF				×			×
GO:0000287	Magnesium ion binding	MF					×		×
GO:0015144	Carbohydrate transmembrane transporter activity	MF					×		×
GO:0090599	α-Glucosidase activity	MF		×	×				×
GO:0003855	3-Dehydroquinate dehydratase activity	MF			×			×	×
GO:0004764	shikimate 3-dehydrogenase (NADP^+^) activity	MF			×			×	×
GO:0008143	Poly(A) binding	MF						×	×
GO:0070717	Poly-purine tract binding	MF						×	×
GO:0030136	Clathrin-coated vesicle	CC			×		×	×	×
GO:0031312	Extrinsic component of organelle membrane	CC					×	×	×
GO:0031314	Extrinsic component of mitochondrial inner membrane	CC					×	×	×
GO:0012510	*trans*-Golgi network transport vesicle membrane	CC					×		×

Only significant GO terms (*P*-value <0.05) for each gene set are marked by ‘×’. Enriched GO terms for drought CHH-DMRs (far right) were used as a basis for comparison among all gene set enrichments. BP, biological process; CC, cellular component; DMR, differentially methylated region; DR-TE, drought-responsive TE; HDR-TE, highly drought-responsive TE; MITE/DTH, Harbinger-related miniature inverted-repeat transposable element; MF, molecular function; SINE, short interspersed nuclear element; TE, transposable element.

## Discussion

In this study, we aimed to characterize the DNA methylation response to multiple stressors in the clonal tree *Populus nigra* cv. ‘Italica’ using whole-genome bisulfite sequencing. By identifying both generic and stress-specific DNA methylation changes in response to stress exposure, we provide insights into the methylome plasticity of this tree species. Our evaluation of the poplar methylome plasticity upon abiotic and biotic treatments allowed the discovery of multi-stress hotspots that are partially shaping natural methylation variation. Moreover, we identified specific TE superfamilies whose response to drought may have been selected to cope with extreme conditions. Our study underscores the importance of considering multiple stressors in elucidating the DNA methylation landscape and its functional implications in long-lived tree species.

### Global signatures of the poplar methylome response to individual stress treatments

#### Abiotic stresses

The global patterns of DNA hypermethylation after exposure to abiotic stresses substantiates previous studies on *Populus* that have reported global DNA methylation increases after 5 weeks of drought stress in *P. trichocarpa* (Liang *et al.*, 2014) and after 7 d of salt stress in *P. euphratica* (Su *et al.*, 2018). In *P. simonii*, methylation gradually increased during the first 24 h of either cold, heat, salinity, or osmotic stress, and certain enzymes involved in (de)methylation pathways were up/down-regulated in a stress-specific manner (Song *et al.*, 2016). However, the low-resolution methods (HPLC and methylation-sensitive amplification polymorphism) used to quantify DNA methylation did not allow the authors of that study to further investigate methylation at context-specific level. Here, using WGBS data, we were able to determine that genome-wide stress-induced hypermethylation can arise in a sequence context specific manner as a response to specific stresses. For instance, we could differentiate CHH hypermethylation induced by drought from CpG/CHG hypermethylation induced by cold (Fig. 2). Together with the mentioned studies, our findings suggest that the context-specific hypermethylation patterns induced by abiotic stresses are the product of the interplay between the establishment of methylation via the RNA-directed DNA methylation pathway and the stress-specific up/down-regulation of demethylation pathways.

Abscisic acid (ABA) is known to initiate stress signaling leading to physiological acclimation upon stress (Popko *et al.*, 2010; Jia *et al.*, 2016, 2017). However, only a few studies have suggested its potential role in mediating global hypermethylation responses (Song *et al.*, 2016; Lafon-Placette *et al.*, 2018; Su *et al.*, 2018). For instance, ABA treatments in Arabidopsis induced hypermethylation at ABA-responsive genes (Gohlke *et al.*, 2013). Moreover, ABA-mediated up-regulation of specific microRNAs can down-regulate targeted demethylases (Sunkar and Zhu, 2004), which in turn may result in hypermethylation. Because increase of methylation may be associated with gene silencing (Paszkowski and Whitham, 2001; Fojtova *et al.*, 2003), stress-induced global hypermethylation may induce progressive gene silencing leading to arrested growth under adverse conditions. However, as some stresses have an effect on tree growth, hypermethylation could also reflect a delayed developmental stage and thus confound the interpretation of the stress-induced methylation change. In this study, we found a large overlap between drought- and heat-induced DMRs (Supplementary Fig. S13). However, reduced growth was observed only after drought, but not after heat treatment (Supplementary Fig. S5). The fact that similar DMRs are induced to different stresses irrespective of a stress effect on growth suggests that many drought-DMRs are probably not a consequence of developmental delay after drought.

In *P. tremula* and tree peony, bud growth reactivation is preceded by a progressive reduction of genomic DNA methylation (Conde *et al.*, 2017; Zhang *et al.*, 2020). Therefore, it is plausible that cold-induced hypermethylation occurs as a first response during winter, arresting growth, followed by a gradual demethylation that leads to growth reactivation in spring. Since changes in CHH methylation are less stable than those in CpG/CHG contexts (Secco *et al.*, 2015; Wibowo *et al.*, 2016), context-specific hypermethylation suggests different durations of the response. This might be related to differences in duration of the environmental stresses in nature, specifically longer cold periods (winter) versus brief episodes of drought during the growing season.

#### Biotic stresses

The effect of biotic stresses on DNA methylation has been examined in Arabidopsis and other species (Dowen *et al.*, 2012; Zhang *et al.*, 2018; Ramos-Cruz *et al.*, 2021), but little information is available about woody plants. It is known that SA treatment, rust infection, and caterpillar attack increase the levels of SA, jasmonic acid, and ABA in the affected poplar leaves (Clavijo McCormick *et al.*, 2014; Eberl, *et al.*, 2018a, b; Li *et al.*, 2018; Ullah *et al.*, 2019). Moreover, SA can be transported from infected to uninfected sites to induce systemic acquired resistance (Li *et al.*, 2018). Therefore, we will discuss the biotic-induced methylation patterns in the context of systemic acquired resistance as we sampled unaffected leaves that developed during the stress periods.

Consistent with our results, treatment with exogenous SA has been reported to induce DNA hypomethylation in other species, which in turn activates defense-response genes (Dowen *et al.*, 2012; Kiselev *et al.*, 2013, 2015; Ngom *et al.*, 2017). The CpG/CHG hypomethylation patterns that we observed upon both herbivory and SA treatment (Fig. 2B) might therefore be related to activating SA-dependent defense response.

Diminished rust infection has been observed in drought-affected poplars, explained to some extent by increased stomatal closure mediated by ABA (Ullah *et al.*, 2019). Our results suggest that diminished rust infection could also be the result of methylation responses, as rust infection also induced CpG/CHG hypermethylation profiles very similar to those observed under drought and heat, but not cold (Fig. 2B). These similarities suggest a possible overlap between the responses to drought and rust infection, as it has been suggested by results on the poplar apoplast proteome (Pechanova *et al.*, 2010).

### Hotspots of environmentally induced methylation variation

Experiments for studying stress effects on DNA methylation usually analyse DMRs induced by single stresses. By intersecting several DMR sets detected from different stresses, we can discriminate between generic and stress-specific responses. However, while the latter approach can pinpoint multi-stress DMRs (Song *et al.*, 2016), the vast number of genomic regions tested in whole-genome studies usually produce a reduced set with only the strongest responding regions. Thus, the intersection approach will likely underestimate the overlapping responses between different stresses. In this study, after observing that many stress-induced DMRs were found in the same genomic regions when comparing among different DMR sets (Fig. 3B), we interrogated the methylation variation in regions where a DMR was identified (Fig. 6A). We found that CpG/CHG-DMRs often showed a similar response irrespective of treatment, suggesting that much of the stress response in poplar is generic, rather than stress-specific. Based on these observations, we suspect that many of the reported stress-specific DMRs in other species likely also have a multi-stress nature, which would imply a more careful interpretation of DMR results in the future.

Our results resemble the observations of epimutational hotspots in nearly isogenic Arabidopsis lines under greenhouse and natural environmental conditions (Becker *et al.*, 2011; Schmitz *et al.*, 2011; Hagmann *et al.*, 2015). Such epimutation hotspots are characterized by steady-state intermediate methylation levels (Hazarika *et al.*, 2022), which was also observed in the control methylation levels of CG/CHG DMRs (Fig. 6B). However, where the intermediate methylation level of Arabidopsis hotspots is due to sparse cytosine methylation (only a subset of CpGs is methylated), in our case it is the result of individual cytosines being partially methylated. Since such CpG/CHG-DMRs are often located on TE flanking regions (Supplementary Figs S13–S15), we propose that TE-mediated stress-induced (de)methylation is the source of methylation variation on the TE edges, here identified as multi-stress DMRs.

### Stress-induced methylation variation as a source of epialleles under natural conditions

In this clonal system, natural methylation variation can be attributed to spontaneous and environmentally induced variation. Here, we identified many CpG/CHG-DMRs among ortets, which are thought to be mitotically stable and hence clonally transmissible. More interestingly, a large proportion of stress-induced DMRs overlapped with ortet-DMRs (Fig. 6C). Transient, stress-responsive epigenetically labile regions have been identified in other species as overlapping with naturally occurring DMRs (Wibowo *et al.*, 2016; Miryeganeh *et al.*, 2022). This result suggests that at least part of the natural methylation variation of the clonal system at a European scale is induced by differences in environments. Consequently, environment-induced methylation variants in CpG/CHG contexts could be fixed and appear as natural epialleles detectable across the tree lifespan.

In contrast, induced CHH-DMRs showed only a minor overlap with ortet-DMRs, even though such DMRs largely arose in response to drought and heat. This observation supports the idea that CHH methylation variation quickly disappears after the stress is gone, preventing induced CHH-DMRs from persisting as natural epialleles. This capability of CpG/CHG methylation to track long-term environmental variation seems to be supported by recent observations in other trees (Heer *et al.*, 2018; Miryeganeh *et al.*, 2022).

### Functionality of the poplar methylome response to drought

As poplar is a fast-growing riparian tree whose high productivity requires high water availability (Vanden Broeck, 2003; Monclus *et al.*, 2006), methylation responses to drought are potentially relevant for the ecology of this species. Recent reports in the species have found significant genotypic variation for drought tolerance (Viger *et al.*, 2016) as well as for drought escape (Yıldırım and Kaya, 2017). Therefore, efficient fine-tuning of the drought escape and tolerance responses is likely a strong selection pressure in this clonal cultivar, which may have promoted the evolution of DNA-methylation-based regulatory mechanisms.

Even though the study of the functionality of DNA methylation would require at the very least quantification of gene expression, some patterns that we observed in the methylome response to drought suggest a functional consequence. Here, we reported TE-associated CHH hypermethylation mostly in gene flanking regions, which has been also described in *P. trichocarpa* (Liang *et al.*, 2014). Our analysis revealed that the hypermethylation response is enriched in specific TE superfamilies: SINE and MITE/DTH (Fig. 5A). The specificity of this response may be relevant for mediating functional stress responses, as a more specific set of genes may be affected by the hypermethylation response.

TE activity can be triggered by biotic and abiotic stress conditions (Seibt *et al.*, 2016; Lanciano and Mirouze, 2018), which in turn may lead to a rapid and extensive TE amplification followed by inactivity and drift (Jiang *et al.*, 2004). Such is the case for SINEs and MITEs irrespective of their inherent differences: retrotransposons versus DNA transposons, respectively. Both superfamilies are relatively short elements frequently inserted close to and within genes (Seibt *et al.*, 2016; Kögler *et al.*, 2020), likely due to their tendency to integrate in hypomethylated DNA regions (Arnaud *et al.*, 2000). Also, both are preferential targets for *de novo* methylation, which can then spread into flanking sequences and may affect the expression of nearby genes (Arnaud *et al.*, 2000; Chen *et al.*, 2014). TE proximity to genes may suggest that stress-induced TE hypermethylation could be a by-product of highly expressed nearby genes, as previously suggested by Secco *et al.* (2015). However, we observed that CHH hypermethylation occurred irrespective of their distance to genes, indicating that such response may not be a consequence of nearby gene expression.

Hypermethylation of entire TE superfamilies in response to stress has not been previously reported in other species. We found that SINE and MITE/DTH elements were already highly methylated (and presumably silenced) under control conditions. Therefore, as drought seems to reinforce such hypermethylation, we speculate that the selective silencing of these elements might have regulation consequences of nearby drought-responsive genes as hinted by GO enrichment results.

By exposing clonally propagated poplar trees to a panel of different stresses, this study revealed stress-specific epigenetic variation that involves hypermethylation of SINE/MITE elements and CHH-DMRs after drought. Importantly, genes associated with such elements were enriched in general responses to drought (e.g. ABA signaling, protein kinase activity, and response to water deprivation). Thus, to test if DNA methylation is involved in the regulation of such genes, follow-up studies are needed to monitor gene transcription and DNA methylation of those loci prior to and during drought stress.

## Supplementary data

The following supplementary data are available at *JXB* online.

Fig. S1. Stress treatment experimental design.

Fig. S2. Latin square design used for plant allocation on the greenhouse table.

Fig. S3. Soil parameters monitored during the experiment.

Fig. S4. Example of DMR calling using jack-knife approach.

Fig. S5. Boxplots of phenotypic measurements on Lombardy poplar ramets after stress treatments.

Fig. S6. Unsupervised hierarchical clustering analysis for methylation data after stress exposure in the Lombardy poplar.

Fig. S7. Additional informative principal components calculated using methylation data from stress-treated Lombardy poplar ramets.

Fig. S8. Intraclass correlation coefficients (ICC) computed for all ramet pairwise comparisons in the three sequence contexts (CpG, CHG, CHH).

Fig. S9. Characterization of CHH methylation levels within and proximal to transposable elements in drought and control samples.

Fig. S10. Metaplots of CpG, CHG, and CHH methylation level differences (versus control group) within and proximal to gene models and transposable elements.

Fig. S11. Characterization of CpG, CHG, and CHH methylation levels within and proximal to gene models and transposable elements.

Fig. S12. Bar plots of average global methylation (%) for each ortet.

Fig. S13. Upset plot of DMR set intersections between treatments.

Fig. S14. Association of stress-DMRs with the closest TE.

Fig. S15. Methylation analysis of a hyper CG-DMR induced by cold and SA treatment.

Fig. S16. Methylation analysis of a hyper CG-DMR induced by cold, drought and rust infection treatment.

Fig. S17. Boxplots of methylation levels for all TE superfamilies in all sequence contexts.

Fig. S18. Methylation analysis for all SINE elements in 1 kb bins along their distribution over genic regions.

Fig. S19. Methylation analysis for all MITE/DTH elements in 1 kb bins along their distribution over genic regions.

Fig. S20. Heatmap and hierarchical clustering of the average difference methylation levels (compared with control) of the 1728 identified stress-DMRs in the corresponding sequence context.

Fig. S21. Summary of DMRs identified between each pair of ortets on each sequence context.

Table S1. Description and geolocation of the ortets from which ramets were collected.

Table S2. Data filtering and resolution of the methylation analyses.

Table S3. Summary statistics of significant stress-DMRs.

Table S4. Summary of number of significant DMRs identified with the jack-knife approach (JK), Methylkit (M), and the intersection of both datasets.

Table S5. Summary statistics of significant ortet-DMRs.

Table S6. Summary of significant DMRs classified according to the methylation direction compared with control group.

Table S7. Summary of significant DMRs classified according to stress specificity.

Table S8. *Z*-test for proportion of DMRs on different genomic regions (Ha: p1>p2).

Table S9. Contingency tables and independence tests for DMR feature enrichment associated to TEs.

Table S10. Fold enrichment analysis of TE superfamilies targeted by drought-induced CHH DMRs.

Table S11. Summary of ortet-DMRs and the intersection with stress-DMRs.

erae262_suppl_Supplementary_Tables_S1-S11_Figures_S1-S21

## Data Availability

The bisulfite sequencing raw data is deposited in the ENA Sequence Read Archive Repository (www.ebi.ac.uk/ena/) under study accession number: PRJEB51831. Extended methods (sample metadata; sequencing, filtering, and mapping statistics; methylation files for the three contexts; list of annotated differentially methylated regions; TE predictions; custom scripts and other relevant data) are deposited at Zenodo (Peña-Ponton *et al*., 2023; doi: 10.5281/zenodo.8428770).

## Abbreviations

DMR: differentially methylated region
MITE: miniature inverted-repeat transposable element
SA: salicyclic acid
SINE: short interspersed nuclear element
TE: transposable element
WGBS: whole genome bisulfite sequencing.

## References

[CIT0001] Akalin A , Kormaksson M, Li S, Garrett-Bakelman FE, Figueroa ME, Melnick A, Mason CE. 2012. methylKit: a comprehensive R package for the analysis of genome-wide DNA methylation profiles. Genome Biology 13, R87. 23034086 10.1186/gb-2012-13-10-r87 PMC3491415

[CIT0002] Arnaud P , Goubely C, Pélissier T, Deragon JM. 2000. SINE retroposons can be used in vivo as nucleation centers for de novo methylation. Molecular and Cellular Biology 20, 3434–3441. 10779333 10.1128/mcb.20.10.3434-3441.2000 PMC85636

[CIT0003] Ashapkin VV , Kutueva LI, Aleksandrushkina NI, Vanyushin BF. 2020. Epigenetic mechanisms of plant adaptation to biotic and abiotic stresses. International Journal of Molecular Sciences 21, 7457. 33050358 10.3390/ijms21207457 PMC7589735

[CIT0004] Balfagón D , Sengupta S, Gómez-Cadenas A, Fritschi FB, Azad RK, Mittler R, Zandalinas SI. 2019. Jasmonic acid is required for plant acclimation to a combination of high light and heat stress. Plant Physiology 181, 1668–1682. 31594842 10.1104/pp.19.00956 PMC6878009

[CIT0005] Becker C , Hagmann J, Müller J, Koenig D, Stegle O, Borgwardt K, Weigel D. 2011. Spontaneous epigenetic variation in the *Arabidopsis thaliana* methylome. Nature 480, 245–249. 22057020 10.1038/nature10555

[CIT0006] Benjamini Y , Hochberg Y. 1995. Controlling the false discovery rate: a practical and powerful approach to multiple testing. Journal of the Royal Statistical Society Series B: Statistical Methodology 57, 289–300.

[CIT0007] Bewick AJ , Zhang Y, Wendte JM, Zhang X, Schmitz RJ. 2019. Evolutionary and experimental loss of gene body methylation and its consequence to gene expression. G3 9, 2441–2445. 31147389 10.1534/g3.119.400365 PMC6686912

[CIT0008] Boyle EI , Weng S, Gollub J, Jin H, Botstein D, Cherry JM, Sherlock G. 2004. GO::TermFinder—open source software for accessing Gene Ontology information and finding significantly enriched Gene Ontology terms associated with a list of genes. Bioinformatics 20, 3710–3715. 15297299 10.1093/bioinformatics/bth456 PMC3037731

[CIT0009] Chen J , Hu Q, Lu C, Kuang H. 2014. Evolutionary genomics of Miniature Inverted-Repeat Transposable Elements (MITEs) in plants. In: Pontarotti P, ed. Evolutionary biology: genome evolution, speciation, coevolution and origin of life. Cham: Springer International Publishing, 157–168.

[CIT0010] Choudhury FK , Rivero RM, Blumwald E, Mittler R. 2017. Reactive oxygen species, abiotic stress and stress combination. The Plant Journal 90, 856–867. 27801967 10.1111/tpj.13299

[CIT0011] Clavijo McCormick A , Irmisch S, Reinecke A, Boeckler GA, Veit D, Reichelt M, Hansson BS, Gershenzon J, Köllner TG, Unsicker SB. 2014. Herbivore-induced volatile emission in black poplar: regulation and role in attracting herbivore enemies. Plant, Cell & Environment 37, 1909–1923. 10.1111/pce.12287 24471487

[CIT0012] Cochran WG. 1977. Sampling techniques. 3rd edn. New York: John Wiley & Sons.

[CIT0013] Conde D , Le Gac AL, Perales M, Dervinis C, Kirst M, Maury S, González-Melendi P, Allona I. 2017. Chilling-responsive DEMETER-LIKE DNA demethylase mediates in poplar bud break. Plant, Cell & Environment 40, 2236–2249. 10.1111/pce.13019 28707409

[CIT0014] Dancho M. 2022. R package tidyquant: Tidy Quantitative Financial Analysis (R package version 1.0.5). https://cran.r-project.org/web/packages/tidyquant/index.html

[CIT0015] Díez-Rodríguez B , Peña-Ponton C, Pérez-Bello P, et al. 2022. An uncommon garden experiment: microenvironment has stronger influence on phenotypic variation than epigenetic memory in the clonal Lombardy poplar. BioRxiv 2022.03.22.485169 [Preprint].

[CIT0016] do Amaral MN , Auler PA, Rossatto T, Barros PM, Oliveira MM, Braga EJB. 2020. Long-term somatic memory of salinity unveiled from physiological, biochemical and epigenetic responses in two contrasting rice genotypes. Physiologia Plantarum 170, 248–268. 32515828 10.1111/ppl.13149

[CIT0017] Dowen RH , Pelizzola M, Schmitz RJ, Lister R, Dowen JM, Nery JR, Dixon JE, Ecker JR. 2012. Widespread dynamic DNA methylation in response to biotic stress. Proceedings of the National Academy of Sciences, USA 109, E2183–E2191. 10.1073/pnas.1209329109 PMC3420206 22733782

[CIT0018] Drost HG , Gabel A, Grosse I, Quint M. 2015. Evidence for active maintenance of phylotranscriptomic hourglass patterns in animal and plant embryogenesis. Molecular Biology and Evolution 32, 1221–1231. 25631928 10.1093/molbev/msv012 PMC4408408

[CIT0019] Dubin MJ , Zhang P, Meng D, et al. 2015. DNA methylation in *Arabidopsis* has a genetic basis and shows evidence of local adaptation. eLife 4, e05255. 25939354 10.7554/eLife.05255 PMC4413256

[CIT0020] Eberl F , Hammerbacher A, Gershenzon J, Unsicker SB. 2018a. Leaf rust infection reduces herbivore-induced volatile emission in black poplar and attracts a generalist herbivore. New Phytologist 220, 760–772. 28418581 10.1111/nph.14565

[CIT0021] Eberl F , Perreca E, Vogel H, Wright LP, Hammerbacher A, Veit D, Gershenzon J, Unsicker SB. 2018b. Rust infection of black poplar trees reduces photosynthesis but does not affect isoprene biosynthesis or emission. Frontiers in Plant Science 9, 1733. 30538714 10.3389/fpls.2018.01733 PMC6277707

[CIT0022] Falcon S , Gentleman R. 2008. Hypergeometric testing used for gene set enrichment analysis. In: Hahne F, Huber W, Gentleman R, Falcon S, eds. Bioconductor case studies. New York: Springer, 207–220.

[CIT0023] Fichman Y , Mittler R. 2020. Rapid systemic signaling during abiotic and biotic stresses: is the ROS wave master of all trades? The Plant Journal 102, 887–896. 31943489 10.1111/tpj.14685

[CIT0024] Fleiss JL , Cohen J. 1973. The equivalence of weighted Kappa and the intraclass correlation coefficient as measures of reliability. Educational and Psychological Measurement 33, 613–619.

[CIT0025] Fojtova M , Van Houdt H, Depicker A, Kovarik A. 2003. Epigenetic switch from posttranscriptional to transcriptional silencing is correlated with promoter hypermethylation. Plant Physiology 133, 1240–1250. 14551338 10.1104/pp.103.023796 PMC281619

[CIT0026] Gamer M , Lemon J, Fellows I, Singh P. 2019. R package irr: Various Coefficients of Interrater Reliability and Agreement (R package version 0.84.1). https://cran.r-project.org/web/packages/irr/index.html

[CIT0027] Gohlke J , Scholz CJ, Kneitz S, Weber D, Fuchs J, Hedrich R, Deeken R. 2013. DNA methylation mediated control of gene expression is critical for development of crown gall tumors. PLoS Genetics 9, e1003267. 23408907 10.1371/journal.pgen.1003267 PMC3567176

[CIT0028] Gupta BK , Sahoo KK, Ghosh A, Tripathi AK, Anwar K, Das P, Singh AK, Pareek A, Sopory SK, Singla-Pareek SL. 2018. Manipulation of glyoxalase pathway confers tolerance to multiple stresses in rice. Plant, Cell & Environment 41, 1186–1200. 10.1111/pce.12968 28425127

[CIT0029] Hagmann J , Becker C, Müller J, et al. 2015. Century-scale methylome stability in a recently diverged *Arabidopsis thaliana* lineage. PLoS Genetics 11, e1004920. 25569172 10.1371/journal.pgen.1004920 PMC4287485

[CIT0030] Hämälä T , Ning W, Kuittinen H, Aryamanesh N, Savolainen O. 2022. Environmental response in gene expression and DNA methylation reveals factors influencing the adaptive potential of *Arabidopsis lyrata*. eLife 11, e83115. 36306157 10.7554/eLife.83115 PMC9616567

[CIT0031] Hannan Parker A , Wilkinson SW, Ton J. 2022. Epigenetics: a catalyst of plant immunity against pathogens. New Phytologist 233, 66–83. 34455592 10.1111/nph.17699

[CIT0032] Hazarika RR , Serra M, Zhang Z, Zhang Y, Schmitz RJ, Johannes F. 2022. Molecular properties of epimutation hotspots. Nature Plants 8, 146–156. 35087209 10.1038/s41477-021-01086-7 PMC8866225

[CIT0033] Heer K , Ullrich KK, Hiss M, Liepelt S, Schulze Brüning R, Zhou J, Opgenoorth L, Rensing SA. 2018. Detection of somatic epigenetic variation in Norway spruce via targeted bisulfite sequencing. Ecology and Evolution 8, 9672–9682. 30386566 10.1002/ece3.4374 PMC6202725

[CIT0034] Hothorn T , Bretz F, Westfall P. 2008. Simultaneous inference in general parametric models. Biometrical Journal 50, 346–363. 18481363 10.1002/bimj.200810425

[CIT0035] Jansson S , Douglas CJ. 2007. Populus: a model system for plant biology. Annual Review of Plant Biology 58, 435–458. 10.1146/annurev.arplant.58.032806.103956 17280524

[CIT0036] Jia J , Li S, Cao X, Li H, Shi W, Polle A, Liu TX, Peng C, Luo ZB. 2016. Physiological and transcriptional regulation in poplar roots and leaves during acclimation to high temperature and drought. Physiologia Plantarum 157, 38–53. 26497326 10.1111/ppl.12400

[CIT0037] Jia J , Zhou J, Shi W, Cao X, Luo J, Polle A, Luo ZB. 2017. Comparative transcriptomic analysis reveals the roles of overlapping heat-/drought-responsive genes in poplars exposed to high temperature and drought. Scientific Reports 7, 43215. 28233854 10.1038/srep43215 PMC5324098

[CIT0038] Jiang N , Feschotte C, Zhang X, Wessler SR. 2004. Using rice to understand the origin and amplification of miniature inverted repeat transposable elements (MITEs). Current Opinion in Plant Biology 7, 115–119. 15003209 10.1016/j.pbi.2004.01.004

[CIT0039] Jueterbock A , Boström C, Coyer JA, Olsen JL, Kopp M, Dhanasiri AKS, Smolina I, Arnaud-Haond S, Van de Peer Y, Hoarau G. 2020. The seagrass methylome is associated with variation in photosynthetic performance among clonal shoots. Frontiers in Plant Science 11, 571646. 33013993 10.3389/fpls.2020.571646 PMC7498905

[CIT0040] Kiselev KV , Tyunin AP, Karetin YA. 2013. Influence of 5-azacytidine and salicylic acid on demethylase gene expression in cell cultures of *Vitis amurensis* Rupr. Acta Physiologiae Plantarum 35, 1843–1851.

[CIT0041] Kiselev KV , Tyunin AP, Karetin YA. 2015. Salicylic acid induces alterations in the methylation pattern of the VaSTS1, VaSTS2, and VaSTS10 genes in *Vitis amurensis* Rupr. cell cultures. Plant Cell Reports 34, 311–320. 25420769 10.1007/s00299-014-1708-2

[CIT0042] Kögler A , Seibt KM, Heitkam T, Morgenstern K, Reiche B, Brückner M, Wolf H, Krabel D, Schmidt T. 2020. Divergence of 3ʹ ends as a driver of short interspersed nuclear element (SINE) evolution in the Salicaceae. The Plant Journal 103, 443–458. 32056333 10.1111/tpj.14721

[CIT0043] Koo TK , Li MY. 2016. A guideline of selecting and reporting intraclass correlation coefficients for reliability research. Journal of Chiropractic Medicine 15, 155–163. 27330520 10.1016/j.jcm.2016.02.012 PMC4913118

[CIT0044] Kuznetsova A , Brockhoff PB, Christensen RHB. 2017. lmerTest package: tests in linear mixed effects models. Journal of Statistical Software 82, 1–26.

[CIT0045] Lafon-Placette C , Le Gac AL, Chauveau D, et al. 2018. Changes in the epigenome and transcriptome of the poplar shoot apical meristem in response to water availability affect preferentially hormone pathways. Journal of Experimental Botany 69, 537–551. 29211860 10.1093/jxb/erx409

[CIT0046] Lanciano S , Mirouze M. 2018. Transposable elements: all mobile, all different, some stress responsive, some adaptive? Current Opinion in Genetics & Development 49, 106–114. 29705597 10.1016/j.gde.2018.04.002

[CIT0047] Larchevêque M , Maurel M, Desrochers A, Larocque GR. 2011. How does drought tolerance compare between two improved hybrids of balsam poplar and an unimproved native species? Tree Physiology 31, 240–249. 21444373 10.1093/treephys/tpr011

[CIT0048] Law JA , Jacobsen SE. 2010. Establishing, maintaining and modifying DNA methylation patterns in plants and animals. Nature Reviews. Genetics 11, 204–220. 10.1038/nrg2719 PMC3034103 20142834

[CIT0049] Li Y , Zhang W, Dong H, Liu Z, Ma J, Zhang X. 2018. Salicylic acid in *Populus tomentosa* is a remote signalling molecule induced by *Botryosphaeria dothidea* infection. Scientific Reports 8, 14059. 30232461 10.1038/s41598-018-32204-9 PMC6145909

[CIT0050] Liang D , Zhang Z, Wu H, et al. 2014. Single-base-resolution methylomes of populus trichocarpa reveal the association between DNA methylation and drought stress. BMC Genetics 15 (Suppl 1), S9. 10.1186/1471-2156-15-S1-S9 PMC4118614 25080211

[CIT0051] Liu J , He Z. 2020. Small DNA methylation, big player in plant abiotic stress responses and memory. Frontiers in Plant Science 11, 595603. 33362826 10.3389/fpls.2020.595603 PMC7758401

[CIT0052] López ME , Roquis D, Becker C, Denoyes B, Bucher E. 2022. DNA methylation dynamics during stress response in woodland strawberry (*Fragaria vesca*). Horticulture Research 9, uhac174. 36204205 10.1093/hr/uhac174 PMC9533225

[CIT0053] López Sánchez A , Pascual-Pardo D, Furci L, Roberts MR, Ton J. 2021. Costs and benefits of transgenerational induced resistance in Arabidopsis. Frontiers in Plant Science 12, 644999. 33719325 10.3389/fpls.2021.644999 PMC7952753

[CIT0054] McGraw KO , Wong SP. 1996. Forming inferences about some intraclass correlation coefficients. Psychological Methods 1, 30–46.

[CIT0055] McNemar Q. 1947. Note on the sampling error of the difference between correlated proportions or percentages. Psychometrika 12, 153–157. 20254758 10.1007/BF02295996

[CIT0056] Miryeganeh M , Marlétaz F, Gavriouchkina D, Saze H. 2022. *De novo* genome assembly and *in natura* epigenomics reveal salinity-induced DNA methylation in the mangrove tree *Bruguiera gymnorhiza*. New Phytologist 233, 2094–2110. 34532854 10.1111/nph.17738 PMC9293310

[CIT0057] Monclus R , Dreyer E, Villar M, Delmotte FM, Delay D, Petit JM, Barbaroux C, Le Thiec D, Bréchet C, Brignolas F. 2006. Impact of drought on productivity and water use efficiency in 29 genotypes of *Populus deltoides* × *Populus nigra*. New Phytologist 169, 765–777. 16441757 10.1111/j.1469-8137.2005.01630.x

[CIT0058] Neves DM , Almeida LAH, Santana-Vieira DDS, Freschi L, Ferreira CF, Soares Filho WS, Costa MGC, Micheli F, Coelho Filho MA, Gesteira AS. 2017. Recurrent water deficit causes epigenetic and hormonal changes in citrus plants. Scientific Reports 7, 13684. 29057930 10.1038/s41598-017-14161-x PMC5651809

[CIT0059] Ngom B , Sarr I, Kimatu J, Mamati E, Kane NA. 2017. Genome-wide analysis of cytosine DNA methylation revealed salicylic acid promotes defense pathways over seedling development in pearl millet. Plant Signaling & Behavior 12, e1356967. 28758879 10.1080/15592324.2017.1356967 PMC5640191

[CIT0060] Nunn A , Can SN, Otto C, Fasold M, Díez-Rodríguez B, Fernández-Pozo N, Rensing SA, Stadler PF, Langenberger D. 2021. EpiDiverse Toolkit: a pipeline suite for the analysis of bisulfite sequencing data in ecological plant epigenetics. NAR Genomics and Bioinformatics 3, lqab106. 34805989 10.1093/nargab/lqab106 PMC8598301

[CIT0061] Nunn A , Rodríguez-Arévalo I, Tandukar Z, et al. 2022. Chromosome-level *Thlaspi arvense* genome provides new tools for translational research and for a newly domesticated cash cover crop of the cooler climates. Plant Biotechnology Journal 20, 944–963. 34990041 10.1111/pbi.13775 PMC9055812

[CIT0062] Paszkowski J , Whitham SA. 2001. Gene silencing and DNA methylation processes. Current Opinion in Plant Biology 4, 123–129. 11228434 10.1016/s1369-5266(00)00147-3

[CIT0063] Pechanova O , Hsu CY, Adams JP, et al. 2010. Apoplast proteome reveals that extracellular matrix contributes to multistress response in poplar. BMC Genomics 11, 674. 21114852 10.1186/1471-2164-11-674 PMC3091788

[CIT0064] Peña-Ponton C , Diez-Rodriguez B, Perez-Bello P, Becker C, McIntyre L, Van der Putten W, De Paoli E, Heer K, Opgenoorth L, Verhoeven KJF. 2023. Data from: High-resolution methylome analysis uncovers stress-responsive genomic hotspots and drought-sensitive TE superfamilies in the clonal Lombardy poplar. Zenodo 8428770. 10.1093/jxb/erae262 PMC11427840 38836523

[CIT0096] Perez-Bello Gil P , De Paoli E. 2023. Gene models for Populus nigra var italica [Dataset]. Zenodo. 10.5281/zenodo.8224032

[CIT0065] Popko J , Hänsch R, Mendel RR, Polle A, Teichmann T. 2010. The role of abscisic acid and auxin in the response of poplar to abiotic stress. Plant Biology 12, 242–258. 20398232 10.1111/j.1438-8677.2009.00305.x

[CIT0066] Raj S , Bräutigam K, Hamanishi ET, Wilkins O, Thomas BR, Schroeder W, Mansfield SD, Plant AL, Campbell MM. 2011. Clone history shapes *Populus* drought responses. Proceedings of the National Academy of Sciences, USA 108, 12521–12526. 10.1073/pnas.1103341108 PMC3145742 21746919

[CIT0067] Ramos-Cruz D , Troyee AN, Becker C. 2021. Epigenetics in plant organismic interactions. Current Opinion in Plant Biology 61, 102060. 34087759 10.1016/j.pbi.2021.102060

[CIT0068] R Core Team. 2022. R: a language and environment for statistical computing. Vienna: R Foundation for Statistical Computing.

[CIT0069] Rood SB , Braatne JH, Hughes FMR. 2003. Ecophysiology of riparian cottonwoods: Stream flow dependency, water relations and restoration. Tree Physiology 23, 1113–1124. 14522717 10.1093/treephys/23.16.1113

[CIT0070] Schmitz RJ , Schultz MD, Lewsey MG, O’Malley RC, Urich MA, Libiger O, Schork NJ, Ecker JR. 2011. Transgenerational epigenetic instability is a source of novel methylation variants. Science 334, 369–373. 21921155 10.1126/science.1212959 PMC3210014

[CIT0071] Schmitz RJ , Schultz MD, Urich MA, et al. 2013. Patterns of population epigenomic diversity. Nature 495, 193–198. 23467092 10.1038/nature11968 PMC3798000

[CIT0072] Schönberger B , Chen X, Mager S, Ludewig U. 2016. Site-dependent differences in DNA methylation and their impact on plant establishment and phosphorus nutrition in *Populus trichocarpa*. PLoS One 11, e0168623. 27992519 10.1371/journal.pone.0168623 PMC5167412

[CIT0073] Secco D , Wang C, Shou H, Schultz MD, Chiarenza S, Nussaume L, Ecker JR, Whelan J, Lister R. 2015. Stress induced gene expression drives transient DNA methylation changes at adjacent repetitive elements. eLife 4, e09343. 26196146 10.7554/eLife.09343 PMC4534844

[CIT0074] Seibt KM , Wenke T, Muders K, Truberg B, Schmidt T. 2016. Short interspersed nuclear elements (SINEs) are abundant in Solanaceae and have a family-specific impact on gene structure and genome organization. The Plant Journal 86, 268–285. 26996788 10.1111/tpj.13170

[CIT0075] Seymour DK , Gaut BS. 2020. Phylogenetic shifts in gene body methylation correlate with gene expression and reflect trait conservation. Molecular Biology and Evolution 37, 31–43. 31504743 10.1093/molbev/msz195

[CIT0076] Song Y , Ci D, Tian M, Zhang D. 2014. Comparison of the physiological effects and transcriptome responses of *Populus simonii* under different abiotic stresses. Plant Molecular Biology 86, 139–156. 25002226 10.1007/s11103-014-0218-5

[CIT0077] Song Y , Ci D, Tian M, Zhang D. 2016. Stable methylation of a non-coding RNA gene regulates gene expression in response to abiotic stress in *Populus simonii*. Journal of Experimental Botany 67, 1477–1492. 26712827 10.1093/jxb/erv543

[CIT0078] Sow MD , Le Gac AL, Fichot R, et al. 2021. RNAi suppression of DNA methylation affects the drought stress response and genome integrity in transgenic poplar. New Phytologist 232, 80–97. 34128549 10.1111/nph.17555

[CIT0079] Storey JD. 2002. A direct approach to false discovery rates. Journal of the Royal Statistical Society Series B: Statistical Methodology 64, 479–498.

[CIT0080] Su Y , Bai X, Yang W, Wang W, Chen Z, Ma J, Ma T. 2018. Single-base-resolution methylomes of *Populus euphratica* reveal the association between DNA methylation and salt stress. Tree Genetics & Genomes 14, 86.

[CIT0081] Sunkar R , Zhu JK. 2004. Novel and stress-regulated MicroRNAs and other small RNAs from Arabidopsis. The Plant Cell 16, 2001–2019. 15258262 10.1105/tpc.104.022830 PMC519194

[CIT0082] Suzuki N , Rivero RM, Shulaev V, Blumwald E, Mittler R. 2014. Abiotic and biotic stress combinations. New Phytologist 203, 32–43. 24720847 10.1111/nph.12797

[CIT0083] Tuskan GA , DiFazio S, Jansson S, et al. 2006. The genome of black cottonwood, *Populus trichocarpa* (Torr. & Gray). Science 313, 1596–1604. 16973872 10.1126/science.1128691

[CIT0084] Ullah C , Tsai CJ, Unsicker SB, Xue L, Reichelt M, Gershenzon J, Hammerbacher A. 2019. Salicylic acid activates poplar defense against the biotrophic rust fungus *Melampsora larici-populina* via increased biosynthesis of catechin and proanthocyanidins. New Phytologist 221, 960–975. 30168132 10.1111/nph.15396 PMC6585937

[CIT0085] Vanden Broeck A. 2003. EUFORGEN Technical Guidelines for genetic conservationand use for black poplar (*Populus nigra*). Maccarese, Italy: International Plant Genetic Resources Institute.

[CIT0086] Viger M , Smith HK, Cohen D, Dewoody J, Trewin H, Steenackers M, Bastien C, Taylor G. 2016. Adaptive mechanisms and genomic plasticity for drought tolerance identified in European black poplar (*Populus nigra* L.). Tree Physiology 36, 909–928. 27174702 10.1093/treephys/tpw017 PMC4969554

[CIT0087] Wibowo A , Becker C, Marconi G, et al. 2016. Hyperosmotic stress memory in Arabidopsis is mediated by distinct epigenetically labile sites in the genome and is restricted in the male germline by DNA glycosylase activity. eLife 5, e13546. 27242129 10.7554/eLife.13546 PMC4887212

[CIT0088] Wu T , Hu E, Xu S, et al. 2021. clusterProfiler 4.0: a universal enrichment tool for interpreting omics data. The Innovation 2, 100141. 34557778 10.1016/j.xinn.2021.100141 PMC8454663

[CIT0089] Xiao D , Zhou K, Yang X, Yang Y, Ma Y, Wang Y. 2021. Crosstalk of DNA methylation triggered by pathogen in poplars with different resistances. Frontiers in Microbiology 12, 750089. 35027912 10.3389/fmicb.2021.750089 PMC8748266

[CIT0090] Xuan A , Song Y, Bu C, Chen P, El-Kassaby YA, Zhang D. 2020. Changes in DNA methylation in response to 6-benzylaminopurine affect allele-specific gene expression in *Populus tomentosa*. International Journal of Molecular Sciences 21, 2117. 32204454 10.3390/ijms21062117 PMC7139286

[CIT0091] Yıldırım K , Kaya Z. 2017. Gene regulation network behind drought escape, avoidance and tolerance strategies in black poplar (*Populus nigra* L.). Plant Physiology and Biochemistry 115, 183–199. 28376411 10.1016/j.plaphy.2017.03.020

[CIT0092] Zandalinas SI , Fichman Y, Devireddy AR, Sengupta S, Azad RK, Mittler R. 2020. Systemic signaling during abiotic stress combination in plants. Proceedings of the National Academy of Sciences, USA 117, 13810–13820. 10.1073/pnas.2005077117 PMC7306788 32471943

[CIT0093] Zhang H , Lang Z, Zhu JK. 2018. Dynamics and function of DNA methylation in plants. Nature Reviews Molecular Cell Biology 19, 489–506. 29784956 10.1038/s41580-018-0016-z

[CIT0094] Zhang X , Yazaki J, Sundaresan A, et al. 2006. Genome-wide high-resolution mapping and functional analysis of DNA methylation in *Arabidopsis*. Cell 126, 1189–1201. 16949657 10.1016/j.cell.2006.08.003

[CIT0095] Zhang Y , Si F, Wang Y, Liu C, Zhang T, Yuan Y, Gai S. 2020. Application of 5-azacytidine induces DNA hypomethylation and accelerates dormancy release in buds of tree peony. Plant Physiology and Biochemistry 147, 91–100. 31855819 10.1016/j.plaphy.2019.12.010

