# Supplementary material for: High-resolution methylome analysis uncovers stress-responsive genomic hotspots and drought-sensitive transposable element superfamilies in the clonal Lombardy poplar
Source: J Exp Bot. 2024 Jun 5;75(18):5839–56. doi: 10.1093/jxb/erae262 (PMC11427840; doi:10.1093/jxb/erae262)
Supplement: erae262_suppl_Supplementary_Tables_S1-S11_Figures_S1-S21 [file erae262_suppl_supplementary_tables_s1-s11_figures_s1-s21.pdf]

SUPPLEMENTARY DATA

Table S1: Description and geolocation of the ortets from which ramets were collected.

Ortet ID	Latitude	Longitude	European region	Country	Site
**Norway 1**	59.91953	10.77228	North	Norway	Oslo Botanical Garden
**Norway 2**	59.20995	10.94738	North	Norway	Fredrikstad
**Poland**	52.72787	19.11867	Central east	Poland	Fabianci
**Czechia**	50.08719	14.31551	Central east	Czechia	Prague West
**Germany 1**	49.50706	8.30128	Central	Germany	Lambsheim
**Germany 2**	52.70788	13.13274	Central	Germany	Marwitz Ost
**Italy 1**	44.60751	10.97689	South	Italy	San Damaso
**Italy 2**	44.56590	11.19290	South	Italy	Budrie

Table S2: Data filtering and resolution of the methylation analyses. Sequencing data was filtered before using it as input for each analysis. Depending on the analysis, different resolutions were targeted.

	Genome-wide methylation					Differential methylation	
	**Average global methylation**	**PCA, HC**	**ICC**	**Methylation profiles (genes, TEs)**	**Methylation profiles (scaffold 1)**	**stress-DMR**	**ortet-DMR**
**Samples included in the analysis**	52	52	52	56	56	56	56
**low-coverage cytosines (removed)**	≤ 5	≤ 5	≤ 5	≤ 5	≤ 5	≤ 5	≤ 5
**low-coverage samples (removed)**	4	4	4	0	0	0	0
**Resolution**	Single base	Single base	100-bp bins	Single base	50-kb bins	Single base	Single base

Table S3: Summary statistics of significant stress-DMRs. For each context and stress, DMR length and number of cytosines per DMR were calculated after merging all DMR callings.

context	stress	DMR length (bp)				# cytosines per DMR			
		mean	sd	min	max	mean	sd	min	max
CpG	cold	184.93	109.00	10.00	584.00	16.32	6.26	10.00	38.80
CpG	drought	210.13	142.49	25.00	693.00	14.80	4.61	10.00	30.00
CpG	heat	217.47	159.91	24.00	731.50	15.39	6.68	10.00	43.00
CpG	herbivory	229.04	102.07	96.67	479.00	17.24	6.11	10.00	33.00
CpG	rust	308.51	262.60	42.00	975.33	16.60	7.75	10.00	35.00
CpG	SA	217.68	105.46	34.00	446.00	16.45	6.48	10.00	39.00
CHG	cold	267.69	221.80	51.00	1295.33	19.86	10.79	10.00	59.50
CHG	drought	298.92	216.52	33.00	1573.00	21.36	10.05	10.00	65.00
CHG	heat	235.57	138.46	50.00	643.50	19.53	8.65	10.00	54.50
CHG	herbivory	307.47	185.19	71.00	791.00	25.87	14.57	10.00	65.00
CHG	rust	261.89	145.79	42.50	1044.00	21.74	10.44	10.00	67.00
CHG	SA	341.56	234.66	50.00	1151.00	23.80	10.95	10.00	60.00
CHH	cold	69.12	45.96	24.00	199.00	17.54	7.15	10.00	33.14
CHH	drought	88.18	58.47	16.00	571.00	19.88	10.83	10.00	80.00
CHH	heat	71.69	44.92	21.00	251.00	17.87	7.57	10.00	47.00
CHH	herbivory	108.50	108.19	32.00	185.00	15.00	2.83	13.00	17.00
CHH	rust	85.00	12.12	78.00	99.00	14.58	1.42	13.00	15.75
CHH	SA	29.00	5.66	25.00	33.00	11.00	1.41	10.00	12.00

Table S4: **Summary of number of significant stress-DMRs identified with the jack-knife approach (JK), Methylkit (M), and the intersection of both datasets.** Intersection JK-M refers to the subset of DMRs found by both JK and M approaches. JK verified with M refers to the percentage of JK DMRs found also by M.

TREATMENT	CpG				CHG				CHH			
	JK	intersection JK-M	M	JK verified with M (%)	JK	intersection JK-M	M	JK verified with M (%)	JK	intersection JK-M	M	JK verified with M (%)
cold	63	56	3078	88.89	81	77	4682	95.06	21	16	3766	76.19
drought	76	65	3596	85.53	140	128	7110	91.43	861	823	96777	95.59
heat	57	45	2304	78.95	79	68	4365	86.08	116	108	7698	93.1
herbivory	32	24	2108	75	21	18	2611	85.71	2	2	4060	100
rust	43	31	3151	72.09	77	69	3817	89.61	3	2	2804	66.67
SA	45	29	3246	64.44	79	69	5263	87.34	2	0	2183	0
average				77.48				89.21				71.92

Table S5: Summary statistics of significant ortet-DMRs. For each context, DMR length and number of cytosines per DMR were calculated after merging all DMR callings among ortets.

context	DMR length (bp)				# cytosines per DMR			
	mean	sd	min	max	mean	sd	min	max
CpG	195.90	96.02	10.29	1083.71	10.88	2.59	10.00	94.29
CHG	206.67	120.27	27.00	993.00	13.23	5.74	10.00	126.43
CHH	71.92	44.81	15.00	423.00	14.02	5.14	10.00	40.86

Table S6: Summary of significant stress-DMRs classified according to the methylation direction compared to control group. For each context, percentages (%) refer to the relative amount of DMRs induced by each treatment. Ratios (hypo/hyper) represent the relative amount of hypomethylated DMRs compared to the number of hypermethylated DMRs.

	CpG				CHG				CHH				TOTAL	
	HYPO	HYPER	%	hypo/ hyper	HYPO	HYPER	%	hyper/ hypo	HYPO	HYPER	%	hyper/ hypo	HYPER	HYPO
Cold	9	54	19.94	0.17	3	78	16.98	0.04	10	11	2.09	0.91	143	22
Drought	58	18	24.05	3.22	121	19	29.35	6.37	0	861	85.67	0.00	898	179
Heat	35	22	18.04	1.59	49	30	16.56	1.63	10	106	11.54	0.09	158	94
Herbivory	16	16	10.13	1.00	11	10	4.40	1.10	1	1	0.20	1.00	27	28
Rust	21	22	13.61	0.95	48	29	16.14	1.66	2	1	0.30	2.00	52	71
SA	28	17	14.24	1.65	65	14	16.56	4.64	2	0	0.20	-	31	95
SUM	167	149		1.12	297	180		1.65	25	980		0.03	1309	489
TOTAL	316		100		477		100		1005		100		1798	

Table S7: Summary of significant stress-DMRs classified according to stress specificity. For each context, percentages (%) refer to the relative amount of DMRs induced by single treatments (stress-specific) and several treatments (multi-stress). Ratios (M/S=Multi-stress/Stress-specific) represent the relative amount of multi-stress DMRs compared to the number of stress-specific DMRs.

	CpG			CHG			CHH		
	Specific	Multi-stress	M/S ratio	Specific	Multi-stress	M/S ratio	Specific	Multi-stress	M/S ratio
**Cold**	32 (14.16%)	31	0.97	43 (14.68%)	38	0.88	10 (1.07%)	11	1.10
**Drought**	36 (15.93%)	40	1.11	73 (24.91%)	67	0.92	804 (86.08%)	57	0.07
**Heat**	23 (10.18%)	34	1.48	34 (11.60%)	45	1.32	61 (6.53%)	55	0.90
**Herbivory**	13 (5.75%)	19	1.46	6 (2.05%)	15	2.50	2 (0.21%)	0	0.00
**Rust**	17 (7.52%)	26	1.53	37 (12.63%)	40	1.08	2 (0.21%)	1	0.50
**SA**	21 (9.29%)	24	1.14	34 (11.60%)	45	1.32	2 (0.21%)	0	0.00
**SUM**	142	84 (37.17%)		227	66 (22.53%)		881	53 (5.67%)	
**TOTAL**	226 (100%)			293 (100%)			934 (100%)		

Table S8: Z-test for proportion of stress-DMRs on different genomic regions (Ha: p1 > p2). DMRs from all stress treatments were merged per context.

**CpG**	**#DMR**	**n1**	**p1**	**bp**	**n2 (total bp)**	**p2**	**pooled p**	**z statistic**	**p-value**
upstream 2kb	26	226	0.1150	72912648	417170123	0.1748	0.1748	-2.3646	0.9910
gene body	91	226	0.4027	137945985	417170123	0.3307	0.3307	2.3002	**0.0107***
downstream 2kb	35	226	0.1549	70254286	417170123	0.1684	0.1684	-0.5439	0.7067
Introns	34	226	0.1504	79918939	417170123	0.1916	0.1916	-1.5712	0.9419
Exons	73	226	0.3230	58082296	417170123	0.1392	0.1392	7.9807	**<0.001***
TE	142	226	0.6283	136755244	417170123	0.3278	0.3278	9.6237	**<0.001***
Intergenic	85	226	0.3761	178172862	417170123	0.4271	0.4271	-1.5497	0.9394
Genic	141	226	0.6239	238997261	417170123	0.5729	0.5729	1.5497	0.0606

**CHG**	**#DMR**	**n1**	**p1**	**bp**	**n2 (total bp)**	**p2**	**pooled p**	**z statistic**	**p-value**
upstream 2kb	21	267	0.0787	72912648	417170123	0.1748	0.1748	-4.1359	1.0000
gene body	74	267	0.2772	137945985	417170123	0.3307	0.3307	-1.8588	0.9685
downstream 2kb	32	267	0.1199	70254286	417170123	0.1684	0.1684	-2.1202	0.9830
Introns	43	267	0.1610	79918939	417170123	0.1916	0.1916	-1.2674	0.8975
Exons	45	267	0.1685	58082296	417170123	0.1392	0.1392	1.3834	0.0833
TE	198	267	0.7416	136755244	417170123	0.3278	0.3278	14.4026	**<0.001***
Intergenic	147	267	0.5506	178172862	417170123	0.4271	0.4271	4.0784	**<0.001***
Genic	120	267	0.4494	238997261	417170123	0.5729	0.5729	-4.0784	1.0000

**CHH**	**#DMR**	**n1**	**p1**	**bp**	**n2 (total bp)**	**p2**	**pooled p**	**z statistic**	**p-value**
upstream 2kb	211	932	0.2264	72912648	417170123	0.1748	0.1748	4.1491	**<0.001***
gene body	162	932	0.1738	137945985	417170123	0.3307	0.3307	-10.1783	1.0000
downstream 2kb	188	932	0.2017	70254286	417170123	0.1684	0.1684	2.7174	**0.0033***
Introns	127	932	0.1363	79918939	417170123	0.1916	0.1916	-4.2905	1.0000
Exons	41	932	0.0440	58082296	417170123	0.1392	0.1392	-8.3986	1.0000
TE	853	932	0.9152	136755244	417170123	0.3278	0.3278	38.2029	**<0.001***
Intergenic	377	932	0.4045	178172862	417170123	0.4271	0.4271	-1.3943	0.9184
Genic	555	932	0.5955	238997261	417170123	0.5729	0.5729	1.3943	0.0816

Table S9: Contingency tables and independence tests for stress-DMRs feature enrichment associated to TEs. Genic region stands for the gene body +/- 2 kb. Each sequence context was tested separately. First, Chi-square tests for independence were performed, then after significant differences were obtained, differences between the marginal proportions (McNemar's test) were also evaluated.

	**CpG**		**CHG**		**CHH**	
	**no TE**	**TE**	**no TE**	**TE**	**no TE**	**TE**
**intergenic**	11	74	20	127	26	351
**Genic region**	73	68	49	71	53	502
Chi-Sqr test		<0.001*		**<0.001***		0.564
McNemar's test		1		**<0.001***		<0.001*
	**no TE**	**TE**	**no TE**	**TE**	**no TE**	**TE**
**intron**	17	17	17	26	10	117
**exon**	46	27	29	16	18	23
Chi-Sqr test		0.654		0.141		**<0.001***
McNemar's test		<0.001*		0.787		**<0.001***
	**no TE**	**TE**	**no TE**	**TE**	**no TE**	**TE**
**upstream 2k**	10	14	6	13	16	194
**gene body**	55	36	37	37	25	137
Chi-Sqr test		0.436		0.559		0.127
McNemar's test		<0.001*		0.001*		**<0.001***
	**no TE**	**TE**	**no TE**	**TE**	**no TE**	**TE**
**downstream 2k**	8	18	6	21	12	171
**gene body**	55	36	37	37	25	137
Chi-Sqr test		0.067		0.100		0.069
McNemar's test		<0.001*		0.048*		**<0.001***

Table S10: Fold enrichment analysis of TE superfamilies targeted by drought-induced CHH DMRs. The total TE length of 136'755.244 bp was used to calculate TE proportions. P-values were adjusted using Bonferroni correction based on the number of tested superfamilies.

**TE superfamily**	**TE length (bp)**	**TE proportion**	**#DMR**	**DMR proportion**	**fold enrichment**	**log2 fold enrichment**	**hypergeometric test (P-value)**	**Adjusted P-value**
DNA/DTA	4160939	0.0304	29	0.0383	1.2574	0.3305	0.127	1.000
DNA/DTC	12503807	0.0914	50	0.0660	0.7214	-0.4710	0.995	1.000
DNA/DTH	3166333	0.0232	27	0.0356	1.5384	0.6215	0.020	0.324
DNA/DTM	16517837	0.1208	57	0.0752	0.6226	-0.6837	1.000	1.000
DNA/DTT	949090	0.0069	10	0.0132	1.9009	0.9267	0.042	0.667
DNA/Helitron	41685589	0.3048	135	0.1781	0.5843	-0.7753	1.000	1.000
LTR/Copia	11836722	0.0866	62	0.0818	0.9450	-0.0816	0.698	1.000
LTR/Gypsy	37558006	0.2746	119	0.1570	0.5716	-0.8068	1.000	1.000
LTR/unknown	12388115	0.0906	69	0.0910	1.0049	0.0070	0.501	1.000
MITE/DTA	668757	0.0049	2	0.0026	0.5396	-0.8902	0.885	1.000
MITE/DTC	172232	0.0013	5	0.0066	5.2376	2.3889	0.003	**0.048***
MITE/DTH	572132	0.0042	27	0.0356	8.5142	3.0899	<0.001	**<0.001***
MITE/DTM	478225	0.0035	3	0.0040	1.1318	0.1786	0.495	1.000
MITE/DTT	61212	0.0004	0	0.0000	0.0000	NULL	1.000	1.000
SINE	471999	0.0035	148	0.1953	56.5712	5.8220	<0.001	**<0.001***
target_site_duplication	2011758	0.0147	15	0.0198	1.3452	0.4278	0.156	1.000

Table S11: Summary of ortet-DMRs and the intersection with stress-DMRs. DMRs were further classified based on its uniqueness, i.e., the frequency they appeared on individual pairwise comparisons: identified on a unique comparison or in more than one comparison.

			stress-DMRs		
**CpG**	**ortet-DMRs**	**%**	**with ortet-DMR intersection**	**%**	**no ortet-DMR intersection**
unique comparison	3044	30.93	21	13.04	
more than one comparison	6796	69.07	140	86.96	
TOTAL	9840		161 (71.24%)		65 (28.76%)
			226 (100%)		

**CHG**	**ortet-DMRs**	**%**	**with ortet-DMR intersection**	**%**	**no ortet-DMR intersection**
unique comparison	1529	20.79	32	11.03	
more than one comparison	5824	79.21	258	88.97	
TOTAL	7353		290 (85.29%)		50 (14.71%)
			340 (100%)		

**CHH**	**ortet-DMRs**	**%**	**with ortet-DMR intersection**	**%**	**no ortet-DMR intersection**
unique comparison	501	43.91	22	36.67	
more than one comparison	640	56.09	38	63.33	
TOTAL	1141		60 (6.36%)		884 (93.64%)
			944 (100%)		

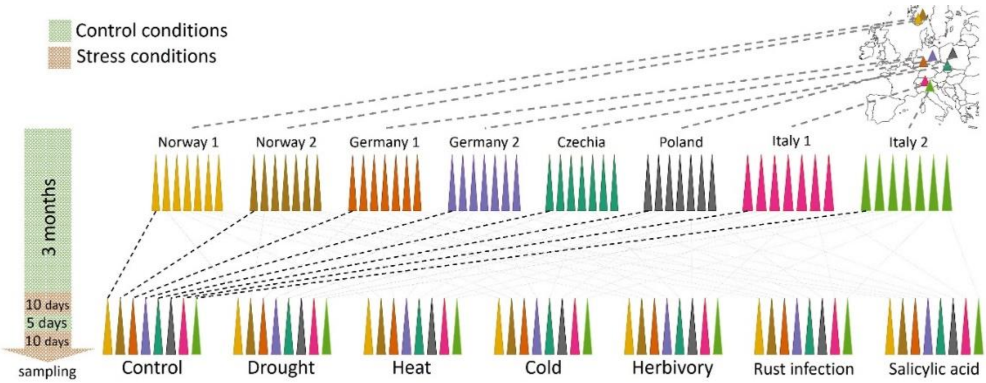

Figure S1: Stress treatment experimental design. Eight adult poplar trees in the field (ortets) were clonally propagated into at least seven ramets per ortet. After 3 months of growth in control conditions*, ramets were exposed to different stress treatments for a total period of 25 days with a recovery period of 5 days in between. At day 26, leaves were sampled for methylation analysis. Different colors represent different ortets. *Temperature: (day/night) 22/18 °C (±2°C), humidity: 60% Rh (±5% Rh), light: (day/night) 16/8 h, VWC: 20.20% (± 3.24 SD)

Ortet ID	Latin square design				Region	Country	
Poland	green	blue	red	yellow	central-east	Poland	Block 1
not sampled	blue	green	yellow	red	central	Germany	
not sampled	yellow	red	blue	green	north	Norway	
Italy 1	red	yellow	green	blue	south	Italy	
Czechia	green	blue	yellow	red	central-east	Czechia	Block 2
Norway 1	yellow	red	blue	green	north	Norway	
Italy 2	red	yellow	green	blue	south	Italy	
Germany 1	blue	green	red	yellow	central	Germany	
not sampled	yellow	red	green	blue	central-east	Poland	Block 3
Germany 2	red	blue	yellow	green	central	Germany	
not sampled	blue	green	red	yellow	south	Italy	
Norway 2	green	yellow	blue	red	north	Norway	

Figure S2: Latin square design used for plant allocation on the greenhouse table. Control (green) and drought-treated (yellow) ramets remained in the same positions during the entire experiment, cold-treated (blue) and heat-treated (red) ramets were allocated on the table only during the stress-free period. Three consecutive blocks of Latin squares were arrayed on a long table ensuring that ramets from every geographic region were included on each block. Ramets from 12 ortets were included in the experiment, however after Allegro genotyping (Díez-Rodríguez et al., 2022), only eight were confirmed to belong to the same clonal lineage, which were sampled for the subsequent analyses shown in this publication. Rust infection, herbivory and SA treatments were implemented in separate greenhouses under the same control growth conditions.

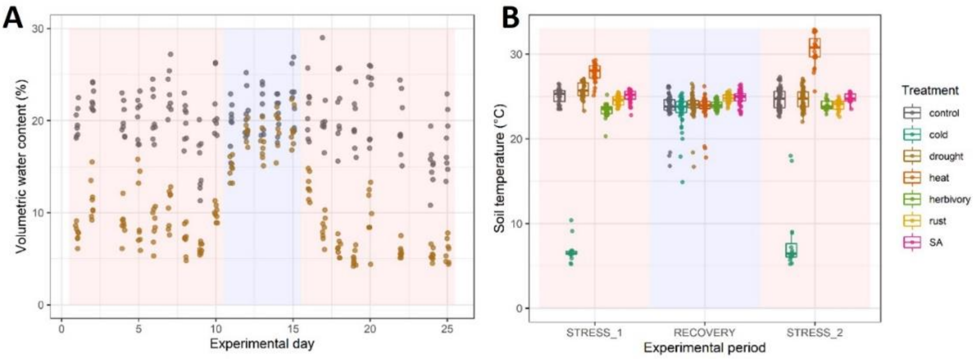

Figure S3: Soil parameters monitored during the experiment. A) Volumetric water content (VWC) is shown for control and drought samples. Two measurements were performed per pot, only the mean VWC is shown. B) Boxplots summarize the soil temperature monitored on each treatment group over the different experimental periods. Stress periods: days 1-10 (STRESS_1), 16-25 (STRESS_2). Recovery periods: days 11-15.

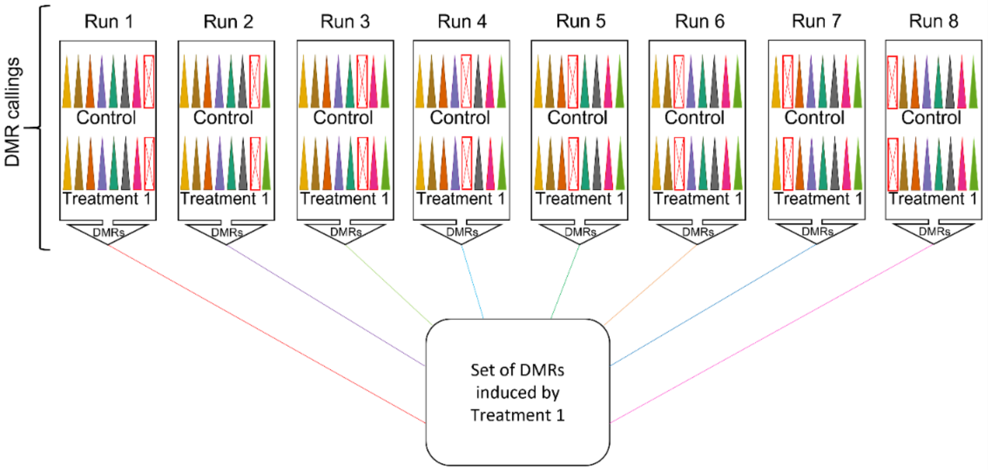

Figure S4: Example of DMR calling using jack-knife approach. For each treatment, eight runs were performed leaving one ortet out on each DMR call. All DMR sets were merged for downstream analysis. Different colors represent different ortets. The red crossed rectangles represent the ortet removed from the respective DMR calling.

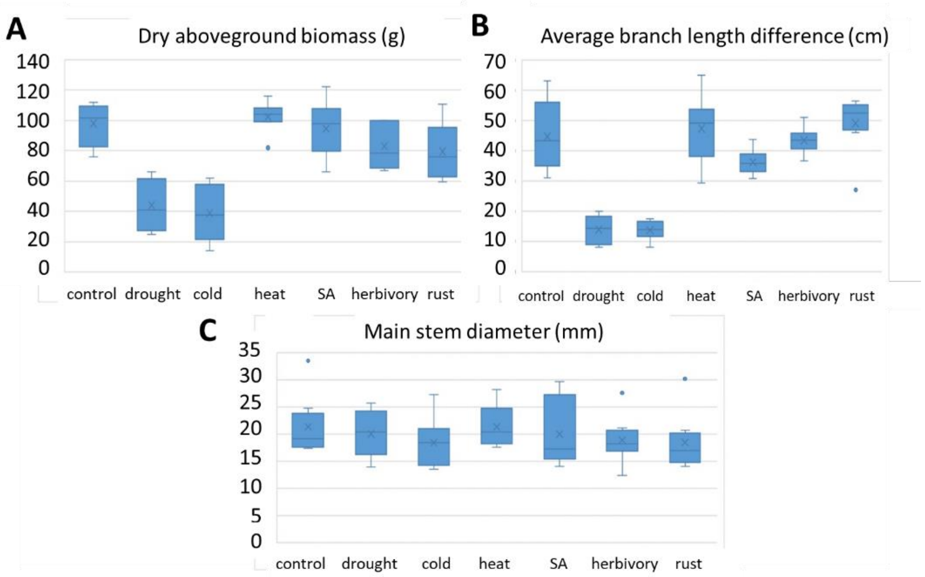

Figure S5: Boxplots of phenotypic measurements on Lombardy poplar ramets after stress treatments. A) Dry aboveground biomass after stress treatment. B) Average branch length difference (experimental day 29 – exp. day 1). C) Main stem diameter. Samples are grouped by treatment.

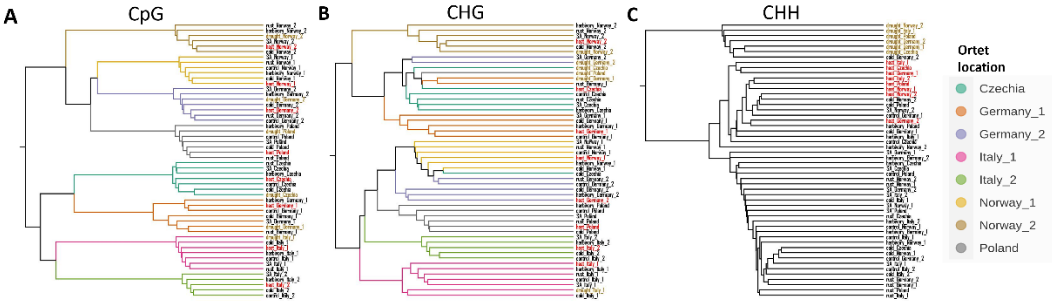

Figure S6: Unsupervised hierarchical clustering analysis for methylation data after stress exposure in the Lombardy poplar: A) CpG, B) CHG and C) CHH methylation. In A) and B), dendrogram branches are colored by ortet location. Some representative nodes (individual samples) are colored according to treatment (drought: brown, and heat: red). For CHH, dendrogram branches were not colored to highlight colors of the nodes.

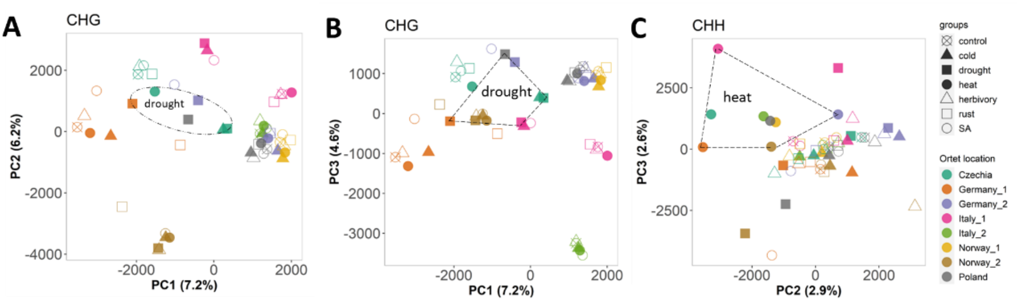

Figure S7: Additional informative principal components calculated using methylation data from stress-treated Lombardy poplar ramets. A) PC1 and PC2 are shown for analysis on CHG context. B) PC1 and PC3 are shown for analysis on CHG context. C) PC2 and PC3 are shown for analysis on CHH context. Samples are colored by ortet identity. Different shapes represent each experimental group. Drought and heat clusters are highlighted within dashed lines.

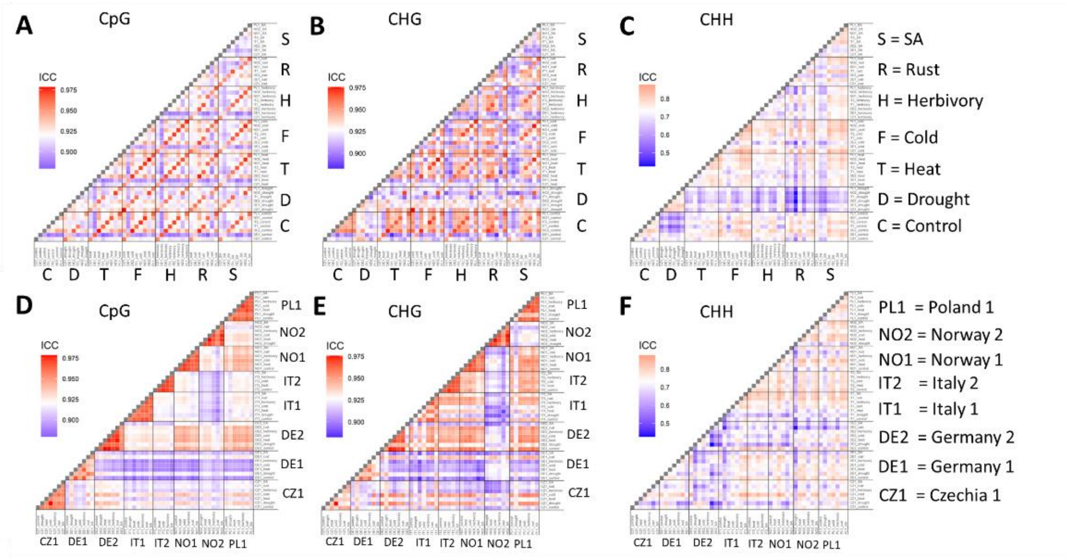

Figure S8: Intraclass correlation coefficients (ICC) computed for all ramet pairwise comparisons in the three sequence contexts (CpG, CHG, CHH). On each plot, each colored tile represents the ICC calculated for the corresponding pairwise comparison. Grids A), B), and C) show samples sorted by stress treatment. Grids D), E), and F) show samples sorted by ortet location. For each plot, the color gradient was determined by maximum (red), minimum (blue) and median (white) ICC values. The methylation level (%) of 27968, 33779 and 125251 100-bp bins were used for ICC calculations for CpG, CHG and CHH contexts, respectively.

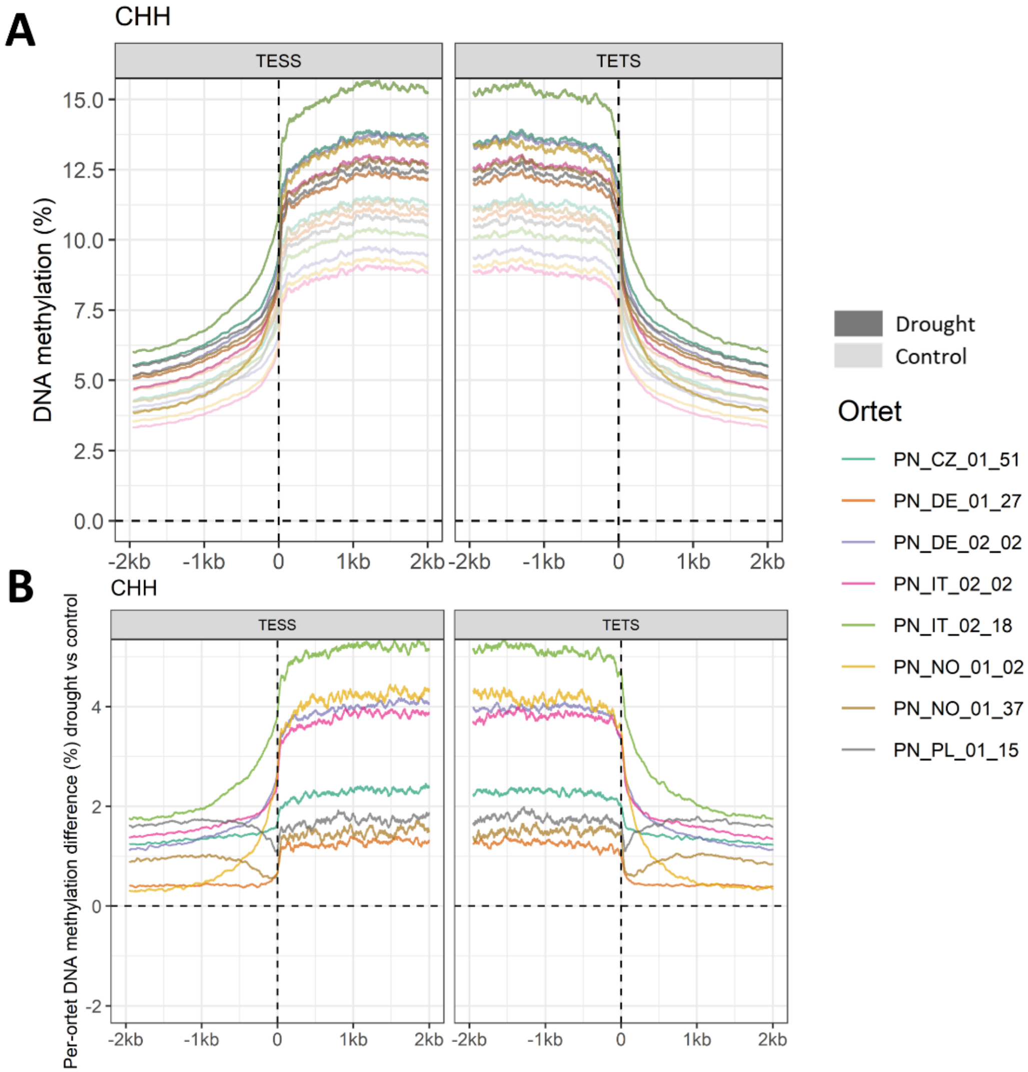

Figure S9: Characterization of CHH methylation levels within and proximal to transposable elements in drought and control samples. A) and B) per-cytosine average methylation was calculated among all transposable elements within each sample according to its relative position to TESS/TETS (see below), A) simple moving averages (SMA) over a period of 50 bp were calculated and plotted for each sample separately. Samples derived from the same ortet are display with similar colors but different intensity (dark: drought, light: control) B) For each ortet, methylation differences were calculated (drought – control) before SMA plotting. Only >200 bp transposable elements were analyzed. The dashed lines represent the points of alignments of annotated transposable elements start site (TESS) and termination site (TETS). For TEs shorter than 2.5kb, relative positions were normalized to 2.5kb.

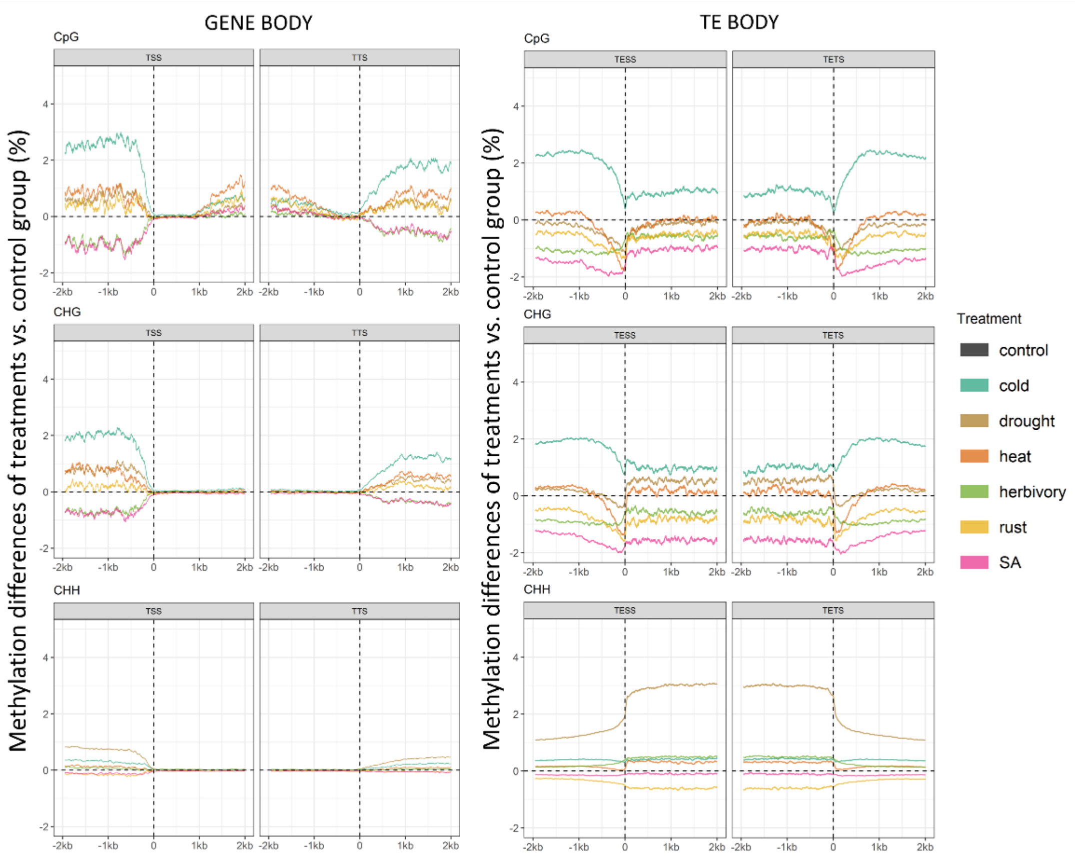

Figure S10: Metaplots of CpG, CHG and CHH methylation level differences (vs control group) within and proximal to gene models and transposable elements. Simple moving averages (SMA) over a period of 50 bp were calculated and plotted for each treatment and context. First, per-cytosine average methylation was calculated among samples within each treatment according to its relative position to TSS/TTS/TESS/TETS (see below), then per-cytosine methylation difference compared to control was calculated and SMA was plotted. Left: methylation differences in gene body and flanking regions. Only protein-coding genes with known 5'UTR and 3'UTR coordinates were analyzed. Right: methylation differences in TE body and flanking regions. Only >200 bp transposable elements were analyzed. The vertical dashed lines represent the points of alignments of coding-gene transcriptional start site (TSS) or annotated transposable elements start site (TESS) and coding-gene transcription termination site (TTS) or annotated transposable element termination site (TETS). Methylation differences refer to differences in percentage points. For TEs shorter than 2.5kb, relative positions were normalized to 2.5kb.

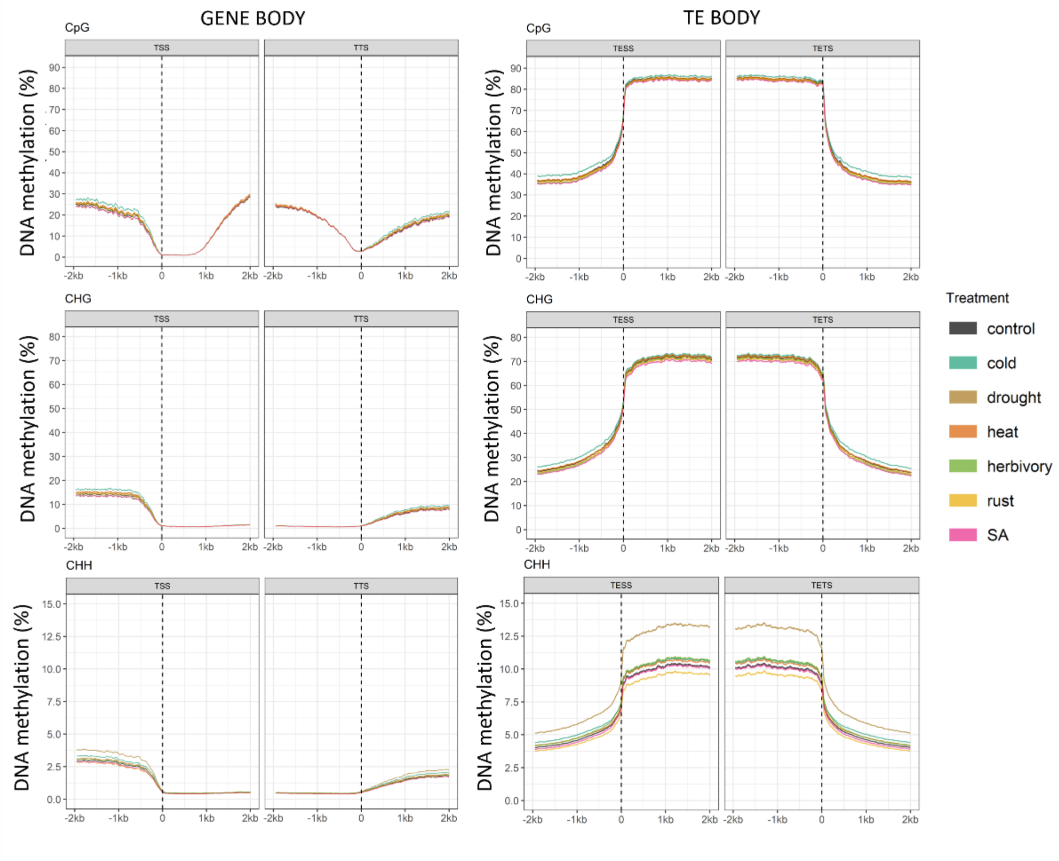

Figure S11: Characterization of CpG, CHG and CHH methylation levels within and proximal to gene models and transposable elements. Simple moving averages (SMA) over a period of 50 bp were calculated and plotted for each treatment and context. First, per-cytosine average methylation was calculated among samples within each treatment according to its relative position to TSS/TTS/TESS/TETS (see below), then SMA were calculated. Left: methylation in gene body and flanking regions. Only protein-coding genes with known 5'UTR and 3'UTR coordinates were analyzed. Right: methylation in TE body and flanking regions. Only >200 bp transposable elements were analyzed. The dashed lines represent the points of alignments of coding-gene transcriptional start site (TSS) or annotated transposable elements start site (TESS) and coding-gene transcription termination site (TTS) or annotated transposable element termination site (TETS). For TEs shorter than 2.5kb, relative positions were normalized to 2.5kb.

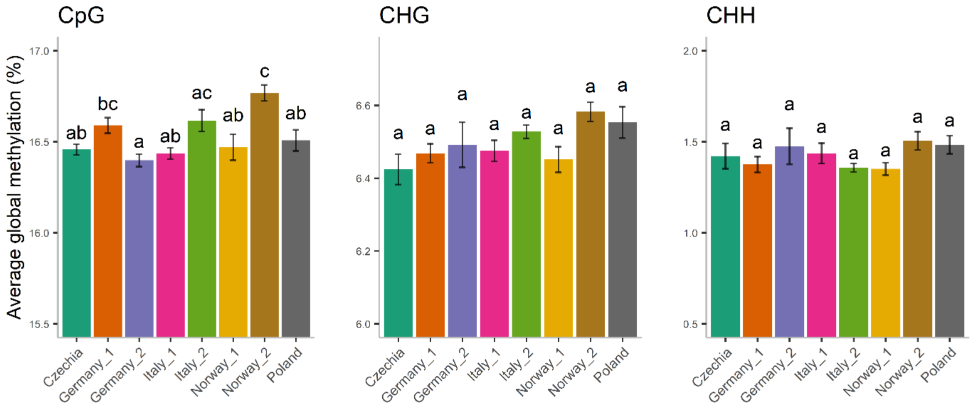

Figure S12: Bar plots of average global methylation (%) for each ortet. Sequence contexts were analyzed separately. Ramets derived from the same ortet and exposed to different stresses were considered as replicates. Horizontal bars and letters indicate relevant significant pairwise differences after Tukey post-hoc comparisons. The values are the means ± s.e.: n=7.

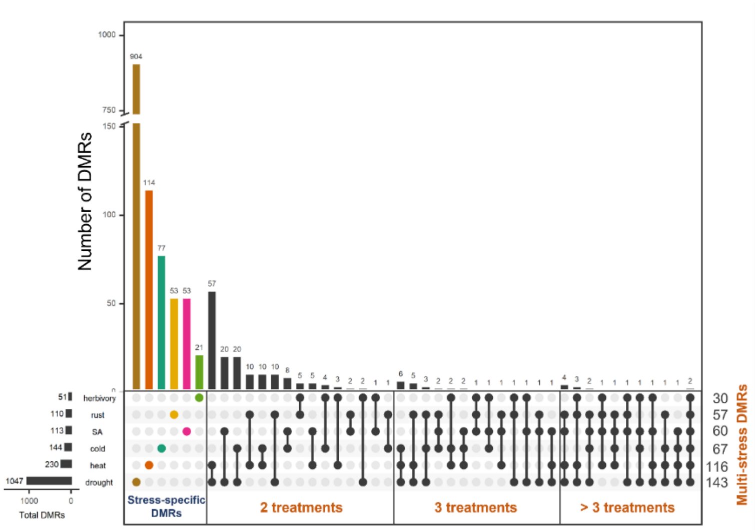

Figure S13: Upset plot of DMR set intersections between treatments. Stress-specific DMRs are shown in colored bars and circles. Multi-stress DMRs are shown in black. DMRs in all sequence contexts were grouped together and methylation direction (hyper/hypo) were ignored for the intersections.

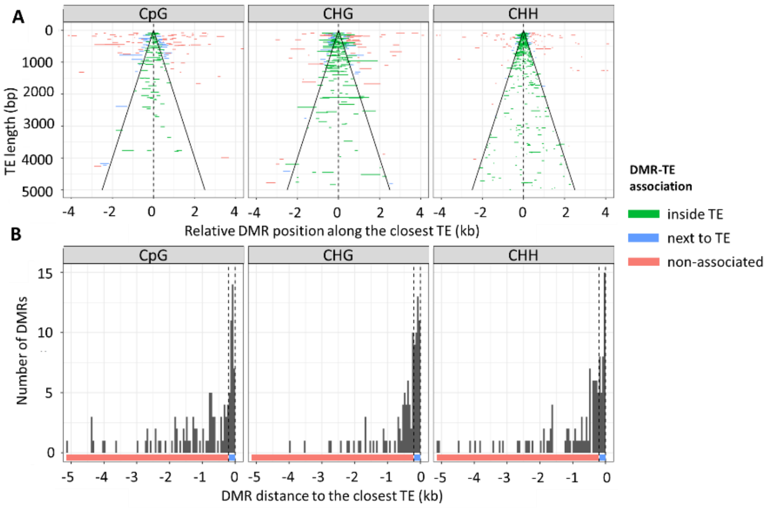

Figure S14: Association of stress-DMRs with the closest TE. A) DMRs (and DMR length) are plotted according to their relative position to the closest TE. TEs are ordered by length (y axis), the relative DMR positions were calculated based on the corresponding TE midpoint (x axis). Sloping lines depict the TE edges. B) Counts of DMRs located close (but not inside) to TEs. DMRs located within the first 200 bp next to a TE were considered as TE-associated. Vertical dashed lines depict the first 200-bp flanking regions of a TE.

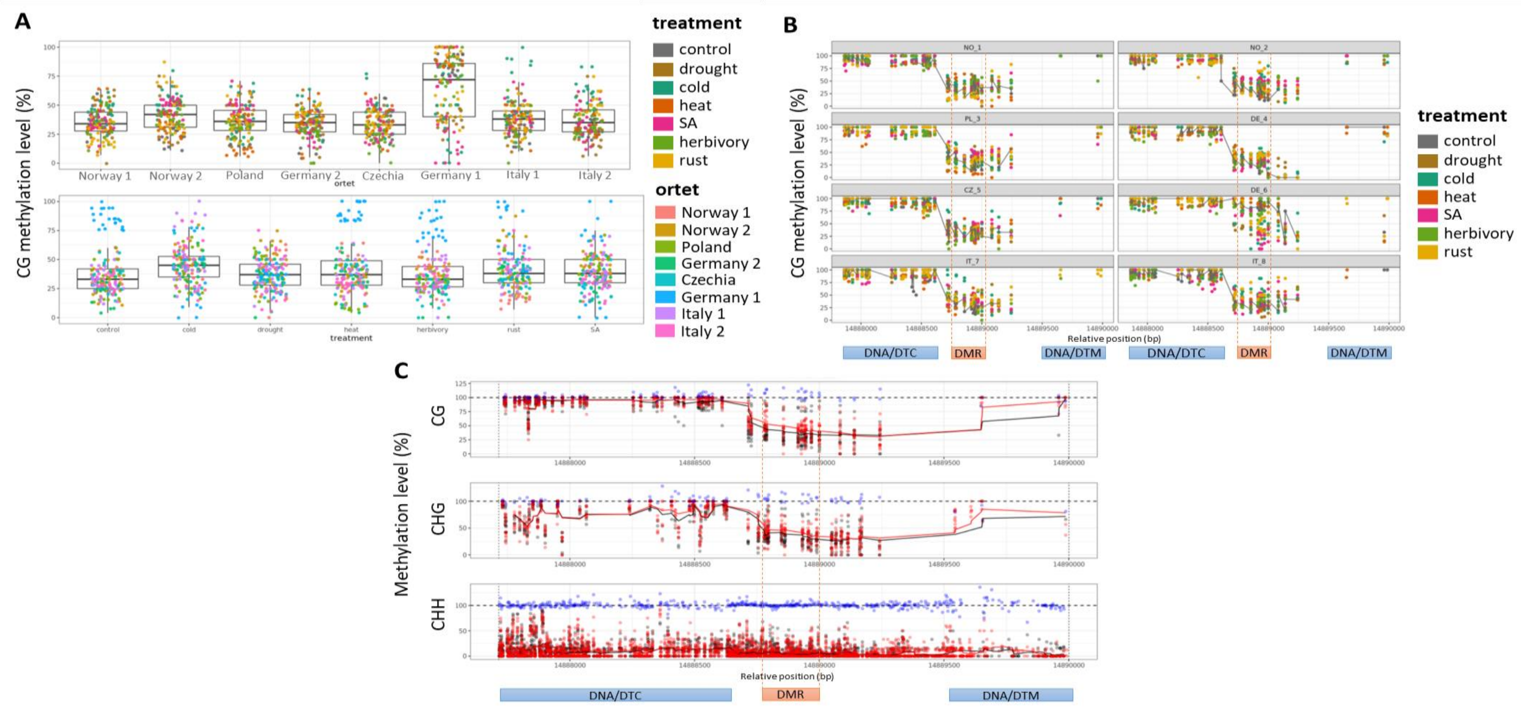

Figure S15: Methylation analysis of a hyper CG-DMR induced by cold and SA treatment. A) Boxplots of the methylation status of all CGs in the DMR, each dot represents a single CG and its methylation status within the DMR. The upper and bottom panels show the variation within ortet and treatment, respectively. B) Each panel shows the methylation profile of the DMR and DMR flanking regions for the respective ortet. Each dot represents a single CG, its methylation status and treatment. C) Each panel shows the methylation profile for each sequence context of the DMR and DMR flanking regions. Each dot represents a single cytosine, its methylation status and treatment (red = cold, black = control). Simple moving averages (SMA) over a period of 5 cytosines are also plotted (red line = cold, black line = control). Blue dots represent the difference between cold and control at each given position but using 100 as base line for better visualization. *The genomic context is shown below the panels: blue and red boxes represent transposable elements and DMR, respectively. Additionally, vertical dotted lines indicate the DMR edges.

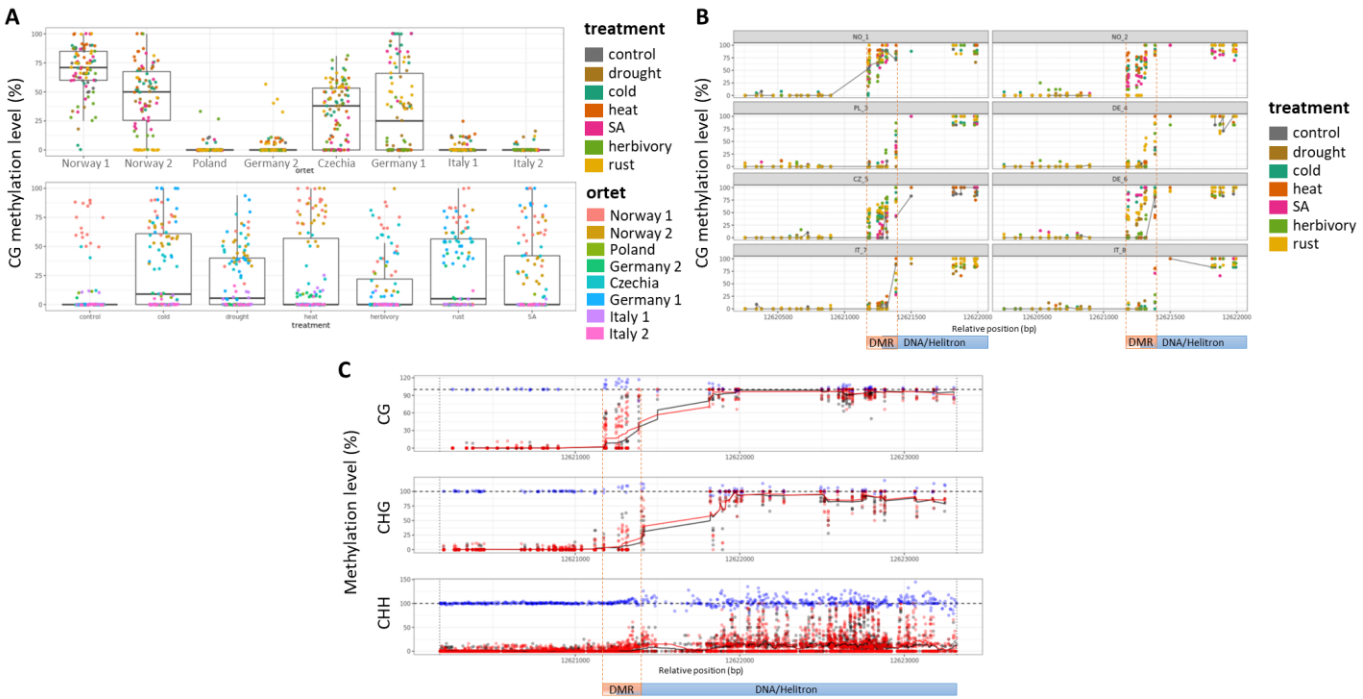

Figure S16: Methylation analysis of a hyper CG-DMR induced by cold, drought and rust infection treatment. A) Boxplots of the methylation status of CGs in the DMR, each dot represents a single CG and its methylation status within the DMR. The upper and bottom panels show the variation within ortet and treatment, respectively. B) Each panel shows the methylation profile of the DMR and DMR flanking regions for the respective ortet. Each dot represents a single CG, its methylation status and treatment. C) Each panel shows the methylation profile for each sequence context of the DMR and DMR flanking regions. Each dot represents a single cytosine, its methylation status and treatment (red = cold, black = control). Simple moving averages (SMA) over a period of 5 cytosines are also plotted (red line = cold, black line = control). Blue dots represent the difference between cold and control at each given position but using 100 as base line for better visualization. *The genomic context is shown below the panels: blue and red boxes represent transposable elements and DMR, respectively. Additionally, vertical dotted lines indicate the DMR edges.

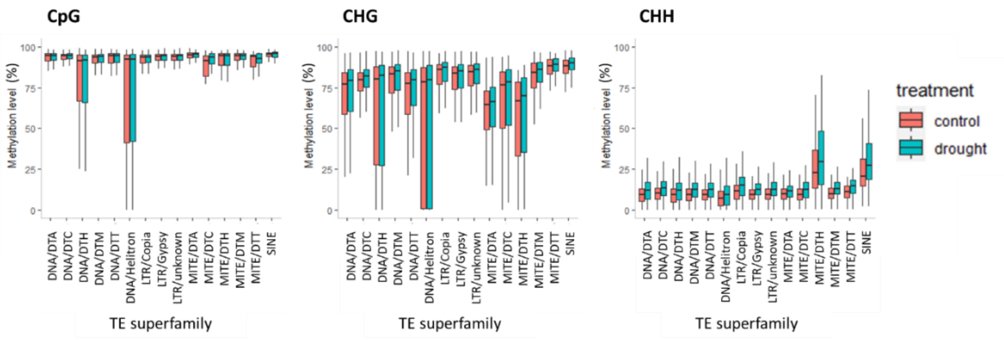

Figure S17: Boxplots of methylation levels for all TE superfamilies in all sequence contexts. Different colors represent control and drought conditions. The average methylation level of each individual transposable

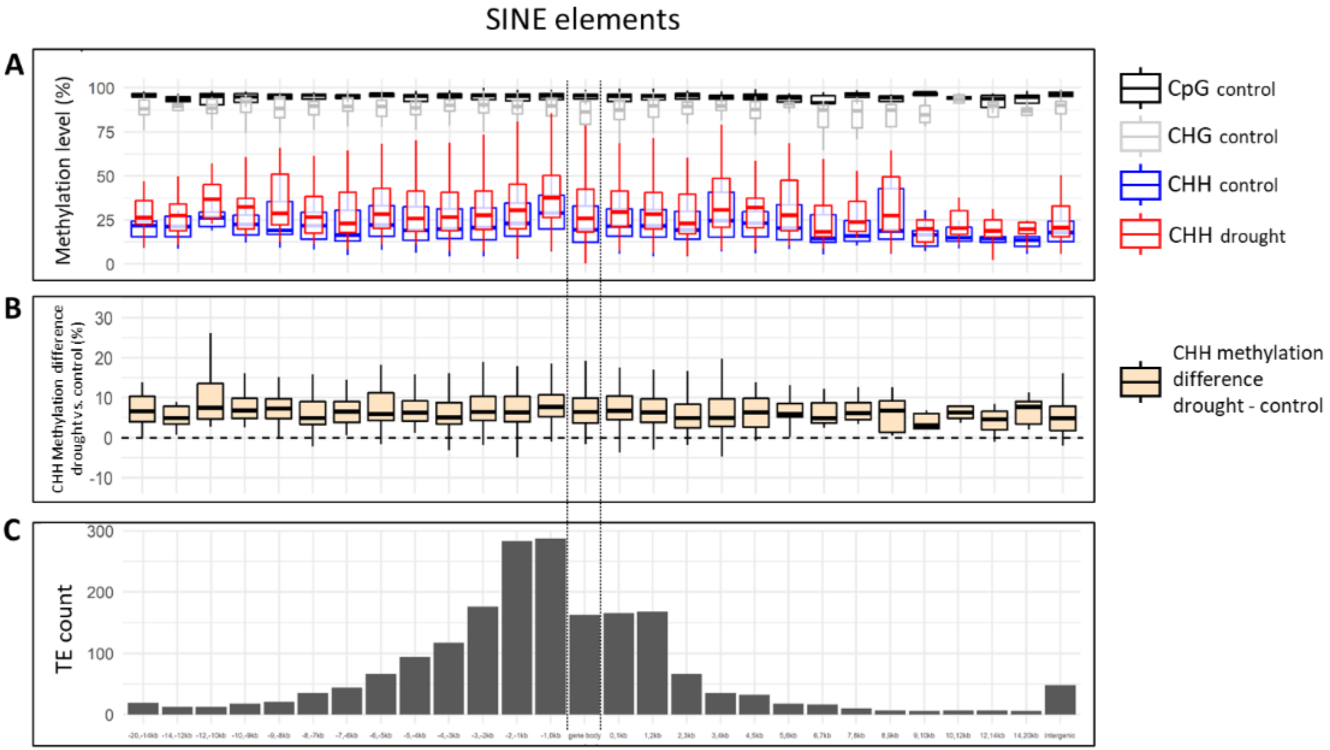

Figure S18: Methylation analysis for all SINE elements in 1-kb bins along their distribution over genic regions. A) Boxplots of the methylation levels of SINE elements for control CG, CHG and CHH methylation, and drought CHH methylation. B) Boxplots of the difference in CHH methylation levels between SINE elements in drought vs. control group. C) Frequency of SINE elements in 1-kb bins along the genic region. All SINEs located inside gene bodies were included in a single bin (doted vertical lines). Elements located more than 10 kb away from the nearest gene were analyzed in 10-12kb, 12-14kb and 14-20kb bins. Intergenic SINEs (far right) correspond to elements located more than 20 kb away from the nearest gene.

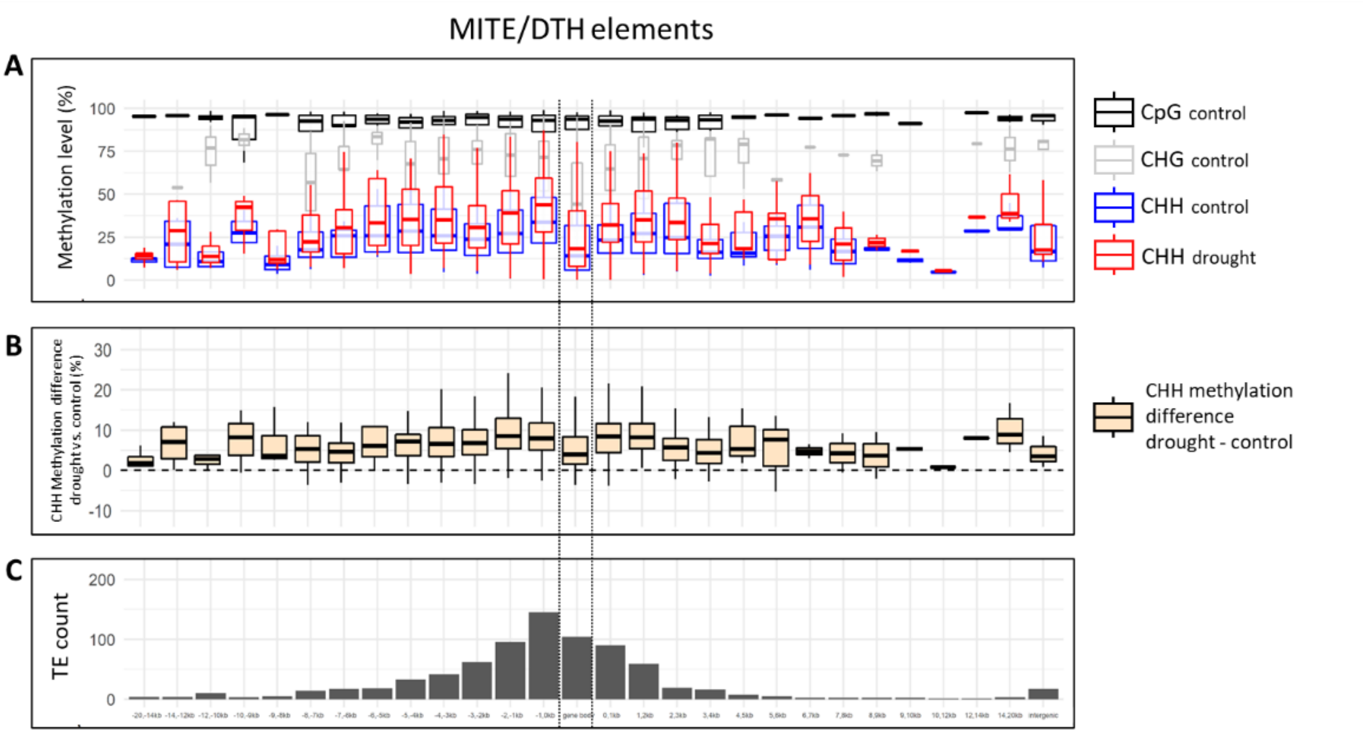

Figure S19: Methylation analysis for all MITE/DTH elements in 1-kb bins along their distribution over genic regions. A) Boxplots of the methylation levels of MITE/DTH elements for control CG, CHG and CHH methylation, and drought CHH methylation. B) Boxplots of the difference in CHH methylation between MITE/DTH elements in drought vs. control group. C) Frequency of MITE/DTH elements in 1-kb bins along the genic region. All MITE/DTH elements located inside gene bodies were included in a single bin (doted vertical lines). Elements located more than 10 kb away from the nearest gene were analyzed in 10-12kb, 12-14kb and 14-20kb bins. Intergenic MITE/DTHs (far right) correspond to elements located more than 20 kb away from the nearest gene.

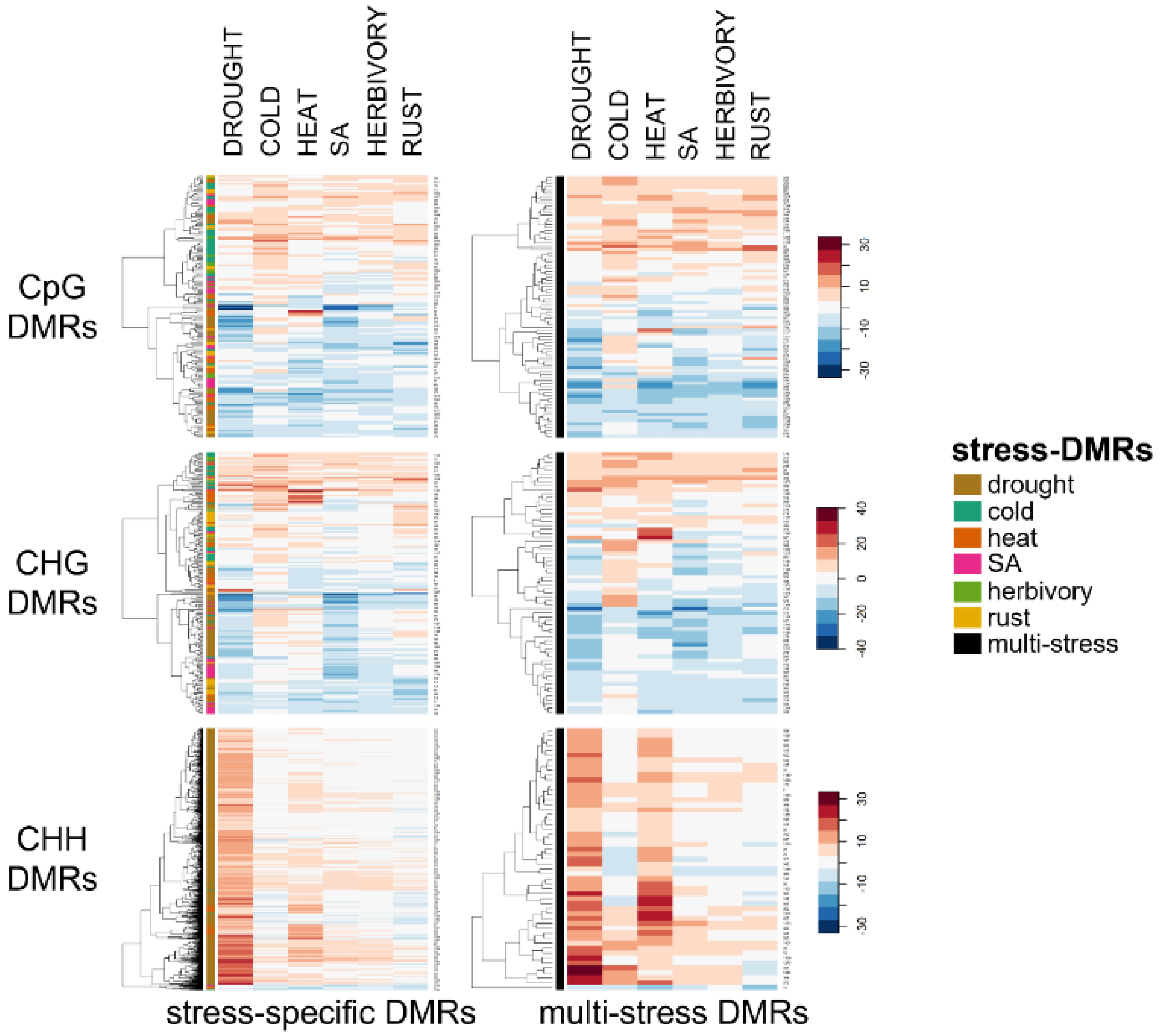

Figure S20: Heatmap and hierarchical clustering of the average difference methylation levels (compared to control) of the 1728 identified stress-DMRs in the corresponding sequence context. Left panel: analysis of DMRs identified in only one stress. Right panel: analysis of DMRs identified in more than one stress.

Figure S21: Summary of DMRs identified between each pair of ortets on each sequence context. Color gradient highlights pairs with the largest differences.

CpG	NO_1	NO_2	PL_3	DE_4	CZ_5	DE_6	IT_7	IT_8	SUM
NO_1		1548	902	818	1274	1762	1529	1358	9191
NO_2	1548		1397	1497	1677	1788	2100	1717	11724
PL_3	902	1397		794	1129	1656	1385	1226	8489
DE_4	818	1497	794		1119	1702	1365	1341	8636
CZ_5	1274	1677	1129	1119		1169	1406	1302	9076
DE_6	1762	1788	1656	1702	1169		1911	1666	11654
IT_7	1529	2100	1385	1365	1406	1911		1380	11076
IT_8	1358	1717	1226	1341	1302	1666	1380		9990
					avg #DMRs per comparison			1425	

CHG	NO_1	NO_2	PL_3	DE_4	CZ_5	DE_6	IT_7	IT_8	SUM
NO_1		2017	1223	946	1782	2129	1305	1266	10668
NO_2	2017		2007	1944	2318	2014	2483	1890	14673
PL_3	1223	2007		1078	1598	1922	1366	1160	10354
DE_4	946	1944	1078		1242	1849	1060	1212	9331
CZ_5	1782	2318	1598	1242		1399	1367	1640	11346
DE_6	2129	2014	1922	1849	1399		1983	1833	13129
IT_7	1305	2483	1366	1060	1367	1983		1368	10932
IT_8	1266	1890	1160	1212	1640	1833	1368		10369
					avg #DMRs per comparison			1621	

CHH	NO_1	NO_2	PL_3	DE_4	CZ_5	DE_6	IT_7	IT_8	SUM
NO_1		226	51	54	69	143	78	58	679
NO_2	226		272	248	191	110	350	270	1667
PL_3	51	272		35	80	254	132	61	885
DE_4	54	248	35		37	187	103	76	740
CZ_5	69	191	80	37		66	92	74	609
DE_6	143	110	254	187	66		179	184	1123
IT_7	78	350	132	103	92	179		60	994
IT_8	58	270	61	76	74	184	60		783
					avg #DMRs per comparison			133	
